# From Genes to Gonads: Molecular Control Points of Reproduction in the Sea Urchin as a Framework for Aquaculture

**DOI:** 10.3390/ijms27198939

**Published:** 2026-10-08

**Authors:** Alfonso Rodríguez, Jorge Olmos

**Affiliations:** Molecular Microbiology Laboratory, Department of Marine Biotechnology, Centro de Investigación Científica y de Educación Superior de Ensenada Baja California, Ensenada 22860, Baja California, Mexico; alfonso.rodriguez@cicese.edu.mx

**Keywords:** sea urchin, gonad development, gametogenesis, sex differentiation, hormone signaling, nutritive phagocytes, nuclear receptors, retinoic acid signaling, metabolomics, aquaculture

## Abstract

The economic output of sea urchin aquaculture depends almost entirely on a single organ. Gonads, marketed as *uni*, determine value through their size, color, texture and flavor, and feed formulations have been optimized empirically for decades with limited reference to the molecular machinery governing gametogenesis, sex differentiation and gonadal maturation in echinoids. This review integrates four lines of mechanistic evidence around the adult gonad, namely developmental gene regulatory networks, sex-biased gonadal transcriptomics, germ-cell and nutritive-phagocyte biology, and endocrine, neuroendocrine and retinoid signaling, and reads them in terms of the control points connecting molecular state to production outcome. Rather than advancing a unified mechanistic model, which the current evidence does not support, each component is classified by the strength of the evidence attached to it: demonstrated in an echinoid, inferred from another taxon, plausible but untested, or hypothetical. The resulting picture is one in which descriptive resources have outpaced functional tests. Candidate sex-determination transcription factors have been identified but none have yet been directly perturbed in an echinoid, the retinoid pathway is architecturally present yet biochemically incomplete, and responses to exogenous steroids remain heterogeneous across species and protocols. Priority experimental directions are ranked by commercial return and by experimental falsifiability.

## 1. Introduction

Sea urchins (Echinoidea) represent a globally traded fishery and aquaculture resource, commanding high prices in East Asian, European and North American seafood markets almost entirely on the basis of their gonads, sold as *uni* or roe. Of the more than 900 echinoid species only less than 10% are considered edible and are commercially exploited, including *Mesocentrotus franciscanus*, *Strongylocentrotus purpuratus*, *S. droebachiensis*, *Loxechinus albus*, *Paracentrotus lividus*, *Echinus esculentus*, *Centrostephanus rodgersii*, *M. nudus*, and *S. intermedius* [1,2,3,4,5,6,7,8,9]. Unlike finfish or crustacean aquaculture the economic output of echinoid culture is determined exclusively by the attributes of the *uni*, making gonadosomatic index (GSI), color, texture, and flavor the critical commercial bottlenecks [1].

Fisheries have traditionally met commercial demand for *uni* through wild capture, harvesting urchins that graze on algal ecosystems as their principal food source [4]. However, the Food and Agriculture Organization (FAO) capture statistics, as compiled in a recent roe-enhancement study, show that global capture of edible sea urchins declined sharply from 109,736 tonnes (live weight) in 1995 to 54,626 tonnes by 2022, a decline the authors attribute to overfishing and the subsequent depletion of native stocks [6]. Within this total, capture of two sea urchins that FAO reports individually, the Chilean sea urchin (*L. albus*) and *Strongylocentrotus* spp., dropped from an annual average of approximately 63,000 tonnes (live weight equivalent) between 2011 and 2020 to 49,000 tonnes in 2022, falling further to 43,000 tonnes by 2024 [10]. In several regions, this decline is further compounded by the formation of urchin barrens—low biodiversity areas dominated by herbivores and deprived of algae. Prolonged nutritional deprivation of sea urchins under barren conditions yields individuals with markedly reduced GSI and diminished pigmentation, rendering them commercially unmarketable irrespective of population abundance [11,12,13].

Addressing the wild capture decline has driven increasing interest in aquaculture-based solutions, particularly full life-cycle culture, aimed at producing market-ready urchins de novo, and short-cycle roe enhancement, whereby wild-harvested urchins from barren grounds are fed formulated diets to restore marketable gonad quality within weeks [1,4,5,11,14]. Many feeds have attempted to yield high quality marketable gonads throughout empirical nutritional trials identifying algae species, carotenoid sources, protein ratios, and fatty acid profile combinations that might enhance gonad index, color, texture and taste [2,4,6,15,16]. However, these dietary advances have developed largely in parallel with, rather than informed by, knowledge of the molecular processes that ultimately determine gonadal phenotype. The machinery governing sex differentiation, gametogenic progression, and endocrine regulation of maturation in echinoids remains only partially resolved.

This mechanistic gap limits the extent to which nutritional and genetic interventions can be rationally designed around defined regulatory targets, rather than empirically iterated through trial-and-error feed formulation. Most of the molecular characterization to date has focused on embryonic and larval stages, where echinoid model species have long served as foundational systems for developmental and evolutionary biology; the adult gonad and its regulatory circuitry remain comparatively underexplored. The mechanistic frameworks most relevant to aquaculture—sex differentiation, gametogenic progression, and gonadal maturation—must often be inferred or extrapolated from developmental datasets rather than drawn from stage-appropriate evidence. In response, four lines of mechanistic evidence bearing on this gap have advanced considerably in recent years, yet largely in isolation from one another, pursued by separate research communities with limited cross-referencing. This review responds to the resulting need for an integrated and updated account, arguing that the available evidence, though fragmentary, is now sufficient to support a structured framework for applied molecular biology in echinoid aquaculture.

First, developmental gene regulatory networks (GRNs) offer a conceptual and analytical framework for echinoid development and reproduction. The embryonic gene regulatory network (GRN) of sea urchins ranks among the most comprehensively characterized transcriptional programs in animal biology, and it supplies a mechanistic vocabulary that can be transposed to the study of post-metamorphic processes, including gonad differentiation and maturation [17,18,19]. Second, gonadal transcriptomes obtained from commercially important species over the last decade have begun to resolve sex-biased gene expression across differentiating and maturing gonadal tissues, identifying candidate sex-determination transcription factors, gametogenesis-associated genes, lipid biosynthesis pathways, and a substantial fraction of still-uncharacterized, species-specific transcripts [20,21,22,23,24,25]. Third, molecular studies of echinoid germ cell biology have established a conserved set of factors governing primordial germ cell (PGC) specification, germ-line stem cell maintenance, and gametogenesis, including germ-line-specific RNA-binding proteins (Vasa, Nanos2), components of the Piwi-interacting RNA (piRNA)–Piwi pathway, and the sexually dimorphic transcription factors *Dmrt1* and *FoxL2* [26,27,28,29]. In parallel, the somatic gonadal compartment dominated by nutritive phagocytes that accumulate nutrients during pre-gametogenic stages and progressively transfer these reserves to developing oocytes and spermatocytes, constitutes the nutritional and structural substrate on which gonad development output ultimately depends [30,31,32,33]. Fourth, and most tentatively, endocrine and retinoic acid (RA) signaling pathways have emerged as candidate regulators of gonadal maturation. Putative roles have been proposed for nuclear receptors (NRs) of the estrogen-related receptor (ERR/NR3B), retinoic acid receptor/retinoid X receptor (RAR/RXR), and steroidogenic (SpSHR2) families, as well as for the RA-catabolizing enzyme cytochrome P450 family 26 (CYP26), although functional evidence in echinoids remains fragmentary. The aromatase-encoding gene *cyp19A* is absent from non-chordate genomes, even in lineages where vertebrate-type sex steroids have been biochemically detected [34]. Figure 1 maps these four lines of evidence onto the germinal and somatic compartments of the adult gonad and onto the commercial attributes of the product, with each molecular component shaded by the class of evidence supporting the function indicated.

Evidence is graded in four classes: A, experimentally demonstrated in an echinoid by functional perturbation or biochemical assay; B, demonstrated in another taxon and inferred for echinoids by orthology; C, plausible inference from expression, genomic presence, or correlation; and D, hypothetical or undemonstrated, including documented absence. The rules by which classes were assigned are stated in Section 8.

Several existing reviews address individual facets of this picture. The determination of the germ line in echinoderm development has been synthesized in detail [35], the evolution of retinoic acid signaling across echinoderms has been reviewed at the genomic level [36], the nutritional strategies underlying gonad enhancement and coloration have been compiled with their ecological context [1], and the endocrine regulation of echinoderm reproduction has been reviewed across the phylum from an evolutionary standpoint [37]; broader treatments of echinoid biology and fisheries are available in edited volumes [31,38]. Each of these, however, addresses a single axis, and most center on embryonic or larval stages or on applied feed formulation rather than on the regulatory biology of the adult gonad. What has been lacking is a thorough synthesis that brings these separate lines together around the adult gonad and reads them in translational terms. The present review is intended to fill that gap: it compiles the developmental, transcriptomic, germ-cell, and endocrine literature into a single account.

Descriptive omics data on echinoids have accumulated rapidly over the last decade, yet functional perturbation experiments remain scarce, and most proposed mechanisms therefore rest on correlation rather than causal test. The scope of this review is deliberately bound. It addresses the molecular biology of the echinoid gonad—the transcriptional programs, signaling pathways, and biochemical circuits that govern its assembly, sexual differentiation, and cyclical alternation between somatic nutrient storage and gametogenesis. Evidence is compiled primarily from *Strongylocentrotus* representatives, extended to other commercially relevant echinoids and, where echinoid data are lacking, supplemented with findings from other echinoderm classes. Rather than advancing a unified mechanistic model, this review is organized around the molecular control points that connect current findings to aquaculture outcomes, distinguishing what is established from what remains to be tested.

This article is a narrative review; therefore, we did not register a protocol, record screening counts or perform a formal quality or risk-of-bias appraisal of individual studies; instead, each line of evidence is graded by the type of assay on which it rests (Section 8). Literature was retrieved from PubMed/MEDLINE, Scopus and Google Scholar, which was used both for keyword searches and for backward and forward citation chaining; Echinobase was used for gene identity and ortholog searches in *S. purpuratus*. The exact search strings were not recorded, and searches in Google Scholar and by citation chaining cannot be reproduced in the same way as database queries. The search was completed in August 2026 and updated with targeted searches during revision in September 2026; thus, statements that a finding has not been reported refer to what these searches identified up to September 2026. Keywords were drawn from four blocks: taxon (echinoid, sea urchin, echinoderm, euechinoid); biological process (gonad, gonadal maturation, gametogenesis, oogenesis, spermatogenesis, sex determination, sex differentiation, nutritive phagocyte, major yolk protein, gonadosomatic index, carotenoid, pigmentation, gonadogenesis); molecular level (transcriptome, RNA-seq, single-cell, proteome, metabolome, methylation, chromatin, microRNA, gene regulatory network, nuclear receptor, retinoic acid, steroidogenesis, neuropeptide); and intervention (knockdown, RNA interference, morpholino, knockout, supplementation, feeding trial). The research window ran mostly from 2000 to 2026, with earlier work recovered by citation chaining, and records published in English or Spanish were considered. Records were kept when they reported molecular, biochemical, genomic or endocrine evidence bearing on the aforementioned blocks, and discarded when echinoids served only as embryo bioassays for ecotoxicological endpoints without gonadal outcomes. Commercial relevance was defined by the existence of a documented capture fishery or roe-enhancement operation. Evidence from non-echinoid echinoderms was admitted only where an echinoid ortholog was documented and classified accordingly in Section 8.

## 2. Gene Regulatory Networks as a Conceptual Framework for Echinoid Reproduction and Gonad Development

### 2.1. Architecture of the Canonical Sea Urchin Embryonic and Larval Gene Regulatory Networks

The GRN directing the partitioning of the sea urchin embryonic endomesoderm into endodermal (gut) and mesodermal (muscle and skeleton) fates stands as a thoroughly validated transcriptional program in animal biology [19]. Constructed over four decades through systematic perturbation analysis and *cis*-regulatory reporter assays in multiple species, the network defines how a small set of maternal transcription factors and signaling molecules progressively partition blastomere lineages into distinct transcriptional states [19,39,40]. This cascade is initiated by the nuclearization of maternal β-catenin at the vegetal pole and propagated through interlocking repression circuits, particularly the double-negative gate, in which Pmar1 represses the repressor HesC to release skeletogenic specification in the micromere lineage, while Delta ligand presented by these micromeres activates Notch signaling in adjacent cells to specify non-skeletogenic mesoderm [41,42,43,44]. A hallmark of this architecture is that such regulatory circuits, that is, the double-negative gate among them, together with positive-feedback loops of the *Otx*–*Brachyury*–*FoxA* type detailed below, lock cells into designated lineages once specification begins, establishing a stable correspondence between lineage and gene expression.

Post-gastrula extensions of the sea urchin GRN have progressively resolved how the regulatory states established in early cleavage give rise to the differentiated cell types of the echinopluteus—or larval—phase, a process now examined at single-cell resolution across developmental stages [45,46]. Following endomesoderm partitioning, the endodermal lineage refines its identity through the same *foxA*–*brachyury* core introduced above, now acting together with *gataE* to stabilize gut specification and pattern the tripartite larval gut into foregut, midgut, and hindgut domains [47]. In parallel, the neurogenic ectoderm gives rise to the ciliary band and to the larval serotonergic and dopaminergic nervous system through a hierarchically organized network: *soxB1* establishes and maintains broad neural progenitor competence, *soxC* drives the progenitor-to-neuron transition, and territorial regulators confer positional identity, together coordinating neuronal differentiation and ciliogenesis [48,49,50,51].

A further particularly well-resolved output of the post-gastrula network is skeletogenesis. The skeletogenic sub-network illustrates how upstream regulatory states converge on effector gene programs. Maternal inputs activate the double-negative gate, in which sequential repression releases downstream skeletogenic activators [43]. This commits the micromere lineage to a skeletogenic fate and drives biomineralization gene batteries through a coherent feedforward cascade [40].

Two features of the post-gastrula GRN literature bear on the sections that follow. First, it demonstrates that the architectural principles of feedback stabilization, gene batteries, and regulatory-state transitions established in the blastula-stage networks extend into progressively differentiated tissues, reinforcing the general applicability of the framework. Second, several of the transcription factor families characterized in these larval contexts (*foxA*, *gata*, and *soxB*) reappear in adult gonadal transcriptomes and in sex-differentiation contexts, providing an ontogenetic anchor for interpreting their expression in reproductive tissues. The broader lesson of these networks, and of the skeletogenic sub-network in particular, is methodological: the sea urchin genome encodes tissue- and stage-specific, feedback-stabilized transcriptional programs that can be resolved to single-gene precision through the same perturbation-based approaches used to build the embryonic network. Applying these approaches to adult gonadal tissue is the central unmet need connecting developmental GRN biology to the reproductive aquaculture context.

### 2.2. Evolutionary Conservation and Diversification of GRNs Across Commercially Relevant Echinoids

Comparative GRN analysis has been a powerful tool for inferring which network elements are ancestral and which are lineage-specific innovations. The foundational comparison of endomesoderm GRNs between the sea urchin *S. purpuratus* and the sea star *Patiria miniata* revealed that a three-gene positive-feedback loop involving *Otx*, *Brachyury*, and *FoxA* has been retained essentially unchanged across echinoderm classes [17,52]. At the same time, comparative studies have documented peripheral rewiring of downstream regulatory connections, particularly in the skeletogenic subcircuit [53]. This rewiring separates the two subclasses of Echinoidea: the earlier-diverging Cidaroidea, which retains ancestral morphological characters [54], and the derived Euechinoidea, which encompasses all commercially exploited species. Cidaroids therefore enter this review as a phylogenetic outgroup, their divergent skeletogenic wiring calibrating which network features are conserved across the class.

The clearest documented diversification of the echinoid developmental GRN comes from comparisons between the cidaroid *Eucidaris tribuloides* and euechinoid species. The two subclasses diverged at least 268 million years ago, and skeletogenic circuitry has been substantially rewired across this period [55]. In *E. tribuloides*, the Pmar1–HesC double-negative gate that specifies euechinoid micromeres is absent; the equivalent segregation of skeletogenic from non-skeletogenic mesoderm proceeds through Delta/Notch signaling instead [53]. Orthologous regulatory factors are nevertheless expressed in corresponding endomesodermal fates in both subclasses, indicating that regulatory-state identity is more evolutionarily stable than the underlying network wiring [56].

Within the echinoid clade, comparative evidence indicates that the developmental GRN is quantitatively conserved across species that share planktotrophic development. Direct comparison of 22–25 developmental regulatory genes—including core endomesoderm regulators such as *otx*, *brachyury*, *foxA*, and *gataE*—between *P. lividus* and *S. purpuratus* revealed near-linear conservation of gene-activation timing, with initiation times more tightly constrained than maximal expression levels [57]. This temporal stability contrasts sharply with the rewiring associated with major life-history transitions. In *Heliocidaris erythrogramma*, the shift from planktotrophic to lecithotrophic development is accompanied by disproportionate transcriptomic change within the GRN itself, exceeding change in the transcriptome as a whole and spanning skeletogenic, endomesodermal, and ectodermal territories [58]. Ecological developmental mode, rather than phylogenetic distance alone, appears to be a principal determinant of GRN expression divergence within echinoids. This evidence comes from *H. erythrogramma*, a lecithotrophic species outside commercial production. The commercially relevant echinoids considered here develop through feeding larvae, as does *S. purpuratus* [59], which favors the transfer of developmental regulatory data among them, although differences in reproductive schedules may still limit it.

The comparative evidence summarized above rests almost entirely on a small number of laboratory model species: *S. purpuratus*, *P. lividus*, *Lytechinus variegatus*, *Hemicentrotus pulcherrimus*, and the two *Heliocidaris* congeners. For the several commercially relevant echinoids that supply most of the gonadal data considered in this review, only *P. lividus* is among them. For *M. franciscanus*, *S. intermedius*, *S. droebachiensis*, *L. albus*, *P. depressus*, *M. nudus*, and *C. rodgersii*, we found no experimental characterization of the developmental GRN as of the search date, and its architecture is inferred by orthology from *S. purpuratus* rather than tested directly. Chromosome-level genome assemblies are now available for *M. franciscanus* [60] and *M. nudus* [61], and comparative single-cell transcriptomics has validated developmental GRN gene deployment in *L. variegatus* at 98% correspondence with the *S. purpuratus* network [62]. No equivalent resource exists for *S. droebachiensis* or *C. rodgersii*. Adult gonadal and sex-differentiation GRNs remain less resolved. Even the underlying mode of sex determination differs between the two species examined and rests on evidence of markedly unequal strength: male heterogamety (XY) was inferred for *P. lividus* from early karyotype analysis [63], whereas female heterogamety (ZW/ZZ) has been independently supported in *M. nudus* by two genomic studies that identified female-specific markers and sex-linked sex-linked single-nucleotide polymorphisms (SNPs) [9,64]. The chromosome-level assembly of *M. nudus* places the sex-linked region on chromosome 7 [61], and its authors read this result as support for rapid evolution of sex-determining mechanisms among sea urchins. Neither system can yet be generalized across echinoid families.

### 2.3. From Larvae to Gonad: The Uncharted Transition

Despite the mechanistic resolution achieved for embryonic and larval GRNs, no experimentally validated GRN for gonadal sex differentiation or gonad development has yet been established in echinoids, and no cross-species functional comparison has been published. What is available is a body of descriptive anatomical, histological, and transcriptomic characterization of the adult gonad, with no perturbation-based dissection of the regulatory logic that produces it. The transcription factors and signaling pathways demonstrated to drive endomesoderm partitioning and skeletogenic specification operate within a narrow embryonic and larval timeframe, and whether any of these factors are re-deployed during post-metamorphic gonad formation has not been directly tested. At the molecular level, it remains unresolved whether the gonad arises from a bipotential primordium that stays sexually labile after metamorphosis, or whether sexual fate is fixed earlier in development; the detection of sex-specific transcription in *L. variegatus* before metamorphosis [22] indicates that divergence begins earlier than the anatomical account implies, without establishing when fate becomes irreversible.

One component of the transition has, by contrast, been characterized in mechanistic detail: the origin of the gonadal primordium itself. During pre-metamorphic development in *S. purpuratus*, the left coelomic pouch gives rise to the genital rudiment, from which the somatic gonadal tissue and the gonoducts subsequently derive; primordial germ cells, set aside earlier from the small micromere lineage, migrate to and colonize this rudiment before metamorphosis [27]. Following metamorphosis, the juvenile gonad develops as five acinar structures radiating from a central genital rhachis, each ultimately draining to the aboral surface through a genital pore [31]. Beyond this anatomical account, however, the molecular events that pattern the primordium, specify its bipotential state, and initiate its sex-specific transcriptional program remain essentially uncharacterized in echinoids.

One approach to this gap has been to ask whether the transcription factors that control gonadal sex differentiation in other animals are also deployed in echinoids. In bilaterians broadly, the antagonistic regulators *Dmrt1* and *FoxL2* constitute a conserved core of the sex-differentiation switch, promoting male and female programs respectively. Sexually dimorphic expression of both has been documented in *M. nudus* gonadal transcriptomes [26,28] and in *L. albus* testis transcriptomes [20], pointing toward regulatory factors whose homologs govern sex differentiation elsewhere; their functional roles in echinoid gonad development, however, have been inferred rather than demonstrated. Similarly, the signaling inputs (Wnt, bone morphogenetic protein (BMP), Hedgehog, fibroblast growth factor (FGF), or otherwise) that might pattern the gonadal primordium and initiate sex-specific transcriptional programs are essentially unknown in echinoids. Recent single-cell characterization of echinoderm ovaries has begun to define the cellular states of the mature gonad across *S. purpuratus*, *L. variegatus*, and *P. miniata* [65], providing a comparative substrate on which future functional GRN work could be built. A companion transcriptomic atlas of adult *L. variegatus* tissues has since resolved the earliest transitions in gonadogenesis, detecting gamete production from approximately 4.5 months post-fertilization and establishing that the ratio of body mass to test diameter predicts sexual maturity more reliably than either age or test size alone [66]. Bridging the embryonic GRN framework to gonad development through systematic perturbation analysis remains one of the central unresolved challenges in echinoid reproductive biology. Resolving how *Dmrt1*, *FoxL2*, and their regulatory inputs specify gonadal sex would also define the molecular levers for directed sex control, though that application depends on first establishing the functional hierarchy among these factors. This framing carries the review from the developmental account of the primordium into the physiological account of the mature gonad that follows.

### 2.4. Gonadal Developmental Stages and Cyclical Reorganization

The mature echinoid gonad occupies a conceptual position distinct from the developmental systems described above. Whereas those networks execute a unidirectional progression from maternal inputs to differentiated cell types, the adult gonad undergoes repeated cyclical transitions between somatic nutrient storage and gametogenesis across the reproductive year [31,67,68]. This distinction raises a question that shapes how the following literature must be interpreted: whether echinoid gonad biology is best framed as a discrete GRN-organized developmental program or as a cyclical physiological transition riding on a stable adult tissue architecture. The available data does not resolve this question, and it is flagged here as an open conceptual issue rather than adjudicated. What can be established descriptively is the tissue architecture on which the cycle operates and the stage-classification system that has been developed to characterize it.

The mature gonad is organized as a compartmental tissue: the acinus is bounded by a germinal epithelium from which the germ-cell lineage arises, and its interior is occupied by nutritive phagocytes among which spermatogenic or oogenic cells develop in male and female gonads, respectively [31,68,69]. This bipartite architecture is conserved across the commercially relevant echinoids that have been examined histologically, including *S. droebachiensis*, *P. lividus*, *M. nudus*, and *L. albus*, indicating that the somatic–germinal partition is a general feature across euechinoids [32,70]. The distinctive feature of the architecture is its seasonal cycling. Outside the reproductive season, the nutritive phagocyte compartment accumulates proteins, lipids, and carotenoids, with the major yolk protein (MYP, a transferrin-family protein) as the dominant proteinaceous reserve [14,33,71]. As gametogenesis initiates, these reserves are progressively transferred to developing oocytes and spermatocytes, and the nutritive phagocyte compartment contracts as the germinal compartment expands [32,33]. The cycle has been subject to multiple staging interpretations. Four stages have been distinguished in *S. droebachiensis* [32], whereas a seven-stage scale has recently been proposed for *P. lividus* [72]. The most widely applied scheme, formalized for *P. lividus* [70] and also used in *S. droebachiensis* [73] and in *P. lividus* from the Aegean Sea [74], recognizes six stages: (I) recovery, (II) growing, (III) pre-mature, (IV) mature, (V) partially spawned, and (VI) spent. This review follows the six-stage classification, and the gonadal stages referred to in the following sections correspond to it. One consequence of this cyclical tissue reorganization is that the same anatomical gonad displays markedly different cellular compositions across the reproductive year, with direct implications for how bulk transcriptomic and biochemical data must be interpreted.

Against this cyclical framework, the most substantial body of molecular data on adult echinoid gonads comes from nutrigenomic studies designed to characterize how dietary composition alters gonadal biology in aquaculture contexts through gonadal transcriptomic and metabolomic profiling. Feeding studies have been performed in *M. nudus*, *P. lividus*, *L. albus*, *S. intermedius*, and *S. purpuratus*, comparing wild-collected animals to those fed formulated or single-source diets [4,11,15,75,76,77,78]. A subset of these studies utilized transcriptomic analyses to identify differentially expressed genes associated with lipid biosynthesis, carotenoid uptake and modification, protein storage, and gametogenic progression [20,24,67,79]. These datasets establish that gonadal transcriptional state is responsive to nutritional inputs across days to weeks, and that key aquaculture-relevant traits—gonad index, coloration, and biochemical composition—correlate with definable transcriptomic shifts.

The interpretive scope of this literature must nonetheless be acknowledged. Most studies were designed to compare diet treatments rather than to test regulatory mechanism, and the correlations they establish between dietary inputs and transcriptomic outputs do not by themselves demonstrate that specific transcription factors, signaling pathways, or epigenetic modifications mediate the observed responses. Because gonadal transcriptomes are stage-dependent, dietary responses detected at one gametogenic stage cannot be assumed to reflect the same regulatory mechanisms as apparently similar responses at another. Nutrigenomic studies therefore characterize adult gonadal transcriptional response at a specific point in the reproductive cycle, rather than the developmental program that produces the mature gonad itself, and translating between the two remains a substantial interpretive leap.

The epigenomic dimension of echinoid gonad biology is considerably less developed. DNA methylation, chromatin accessibility, and non-coding RNA regulation have been characterized in *S. purpuratus* embryos, mainly achieved through comparative methylome analyses [80] and, more recently, integrated scRNA-seq, scATAC-seq, and scSLAM-seq resources spanning cleavage to pluteus stages, currently available as a preprint [81]. Small RNA and piRNA-pathway components have been characterized in echinoid germ cells, principally in *S. purpuratus* [27]. Comparable resources for the adult gonad are unevenly developed. The non-coding RNA layer is the exception: sex-biased microRNAs have been profiled in *M. nudus* gonad by deep sequencing [82], and integrated microRNA–mRNA analysis in *S. intermedius* has linked differentially expressed microRNAs to the carotenoid-uptake machinery discussed in Section 6.3 [24]. Beyond this, however, the chromatin-level resources are absent. No comprehensive DNA methylation atlas, chromatin accessibility profile, or histone modification landscape has been published for the echinoid gonad, and the stage-dependence of these epigenomic states across the reproductive cycle remains unexamined.

This absence constitutes one of the more consequential gaps between the descriptive and transcriptomic data currently available and the mechanistic framework this review anticipates: without adult-tissue, stage-resolved epigenomic characterization, the regulatory logic connecting dietary inputs, transcriptional response, and gonadal developmental outcomes cannot be resolved beyond correlation. Resolving these gaps will ultimately depend on a finer characterization of the two cellular compartments that jointly define the mature gonad: the somatic nutritive phagocytes and the germ line.

## 3. Gonadal Architecture: Cellular and Molecular Foundations

### 3.1. The Dual Cellular Organization: Nutritive Phagocytes and Germ Cells

The mature echinoid gonad is best described as a two-compartment tissue (Figure 2), in which a somatic compartment of nutritive phagocytes provides structural, metabolic, and nutritive support to a germinal lineage that arises from the germinal epithelium at the acinus wall and undergoes gametogenesis within the somatic compartment itself. This organization has been characterized histologically in *S. purpuratus*, *S. droebachiensis*, *P. lividus*, *M. nudus*, and *L. albus*, spanning phylogenetically and geographically distinct euechinoids and indicating that it is not a species-specific arrangement [31,32,69,70]. Terminology for the somatic cell types within this compartment has been inconsistent across the literature: earlier histological studies applied the collective term “accessory cells” to a population that cytochemical studies have since resolved into nutritive phagocytes [28,29] and, adjacent to developing oocytes, a smaller population of follicle cells [83]. Single-cell transcriptomic profiling of echinoderm ovaries has since recovered a *FoxL2*-enriched somatic cluster that its authors assign to follicle cells by analogy with *FoxL2*-expressing granulosa cells of vertebrates [65], and in the sea star *P. miniata*, *Foxl2* transcripts have been localized to follicle cells in situ [84]. In echinoids, this cluster-to-cell-type assignment remains inferential: it rests on marker expression, the *FoxL2*-enriched cluster is also enriched in MYP transcripts, and neither its spatial identity nor its lineage and function have been established. We use the term follicle cells in this sense throughout. Figure 2 summarizes this dual-compartment organization, the reserve-transfer interface between the two compartments, and the current evidence status of the molecular components involved.

The somatic compartment is dominated by nutritive phagocytes, large phagocytic cells that accumulate the biochemical reserves (proteins, lipids, and carotenoids) that fuel gametogenesis and, in oocytes, are transferred to the developing egg as maternal provision [31,32,33]. The dominant proteinaceous reserve is MYP, synthesized primarily in nutritive phagocytes, stored in cytoplasmic granules, and mobilized proteolytically as gametogenesis proceeds; MYP has been characterized biochemically and genetically in *S. purpuratus*, *P. lividus*, and *M. nudus*, and its cyclical accumulation and depletion constitute the biochemical signature of the gonadal cycle [71]. Alongside this proteinaceous store, carotenoid-containing lipid droplets accumulate within the same cells, with 9′-*cis*-echinenone and β-carotene reported as the principal carotenoid species across commercially exploited euechinoids [4,24,67,85,86].

Single-cell transcriptomic profiling of *S. purpuratus* ovaries has resolved 14 distinct cell states, including a *FoxL2*-enriched somatic cluster that is also enriched in MYP and vitellogenin-related transcripts [65]. In situ hybridization in that study localized marker transcripts to early oocytes and the ovarian epithelium, and the correspondence between the remaining somatic clusters and the histologically defined nutritive phagocytes has not been tested [65]. A comparable *FoxL2*-enriched cluster has been recovered not only in the euechinoid *L. variegatus* but also in the asteroid *P. miniata*, a phylogenetic span of roughly 480 million years since the divergence of echinoids and asteroids. Its recovery in all three species suggests that a *FoxL2*-expressing somatic population of the echinoderm gonad may represent an ancestral ground plan of the phylum rather than a lineage-specific arrangement, although functional conservation beyond transcriptional identity remains to be tested.

The germ-cell compartment traces to a small population of primordial germ cells (PGCs) set aside during embryogenesis from the small micromere lineage. Vasa-positive small micromeres migrate to the left coelomic pouch during larval development and colonize the genital rudiment before metamorphosis [27,87]. In the post-metamorphic gonad, these founder cells establish and maintain the germinal epithelium that lines the acinar structures (the gonia visible against the epithelium in Figure 2), and their descendants progress through gametogenesis in a stage-organized manner along the six-stage cycle defined in Section 2.4: recovery (I), growing (II), pre-mature (III), mature (IV), partially spawned (V), and spent (VI). Spermatogenesis proceeds through spermatogonial proliferation, meiotic progression through primary and secondary spermatocytes, and terminal differentiation into spermatids and mature spermatozoa within stages II through IV; oogenesis proceeds through oogonial expansion, primary oocyte growth, vitellogenesis, and terminal maturation across the same stages, with characteristically larger cell dimensions and slower stage progression than the male germ line [32,69]. The functional interface between the two compartments is defined by reserve transfer: as gametogenesis advances from stage II to stage IV, MYP and other reserves accumulated in the nutritive phagocyte compartment are progressively mobilized and transferred to the developing oocytes and, to a lesser extent, spermatocytes, coupling the state of the somatic reserve pool to the biosynthetic demands of the germ line [14,33,71]. This coupling is what makes the somatic-germinal architecture a functional dual system rather than merely a spatial one, and it is the cellular substrate for the molecular events described in Section 3.2.

### 3.2. Molecular Footprint of Germ-Line Differentiation: Vasa, Nanos2, Piwi

The molecular specification and maintenance of the echinoid germ line rest on a small set of experimentally characterized RNA-binding proteins and Argonaute-family effectors that act together as a functional regulatory core across PGC specification, embryonic maintenance, and adult germinal-tissue biology: Vasa, Piwi, and Nanos2. Each is conserved across metazoans and contributes a distinct activity: Vasa regulates the translation of germ-line mRNAs; Piwi suppresses transposons through piRNA-guided silencing; and Nanos2 maintains the undifferentiated germ-line state. The strongest evidence base for all three is derived from *S. purpuratus* embryos and larvae, with adult-tissue functional characterization currently limited to *M. nudus* Nanos2 work.

Vasa is a DEAD-box, ATP-dependent RNA helicase whose canonical function across metazoans is the translational regulation of specific germ-line mRNAs [88,89]. In sea urchins, *vasa* mRNA is deposited maternally and distributed uniformly throughout the early embryo, but Vasa protein accumulation is progressively restricted to the small micromere lineage from the 16-cell stage onward and is maintained specifically in PGC descendants through gastrulation and into larval development [27,87,89]. This translational restriction pattern is characteristic of translational-control mechanisms and implies a substantial regulatory layer between transcript deposition and functional protein expression in the germ-line lineage. Beyond its role as a germ-line marker, Vasa participates directly in the translational regulation of cell-cycle mRNAs in *S. purpuratus* embryos. Specifically, its protein levels oscillate with the cell cycle, and it is associated with the mitotic spindle during metaphase. Furthermore, inhibiting its translation impairs the translation of *cyclinB* mRNA, which disrupts chromosome segregation and delays cell-cycle progression. This cell-cycle role, distinct from its germ-line function, provides one of the clearest examples of a germ-line-specification factor with an experimentally demonstrated biochemical activity in this taxon.

Piwi proteins are members of the Argonaute superfamily of small-RNA-binding effectors and function in most metazoan germ lines through the Piwi-interacting RNA (piRNA) pathway, a small-RNA-guided post-transcriptional and epigenetic silencing system dedicated primarily to the suppression of transposable elements in gonadal tissue [90,91]. The pathway operates by loading Piwi proteins with 24–32-nucleotide piRNAs derived from genomic piRNA clusters; these piRNA–Piwi complexes then guide sequence-specific cleavage of transposon transcripts and, in some contexts, direct heterochromatin formation at transposon loci. In *S. purpuratus*, the Piwi ortholog Seawi is expressed uniformly in the early embryo and becomes enriched in the primordial germ cells from the blastula stage onward, paralleling Vasa and marking the small micromere lineage that is set aside at the fifth cleavage division [27]. Morpholino knockdown of *Sp-seawi* diminishes PGC-specific localization of Seawi protein and dysregulates the expression of Vasa and of Gustavus, the SOCS-box adaptor that controls Vasa protein turnover, while leaving Nanos expression unaffected [27]. The knockdown further induces ectopic proliferation of Vasa-positive cells derived from the small micromere lineage, which subsequently undergo apoptosis by the larval stage; under severe knockdown, apoptosis extends throughout the gut region [27].

Two features of this echinoid characterization warrant emphasis. First, it establishes Piwi as a functional regulator of PGC survival and germ-line marker localization in this taxon, providing a direct precedent for reverse-genetic dissection of echinoid germ-line biology. Second, because *Sp-seawi* knockdown perturbs *vasa* and *gustavus* while sparing *nanos*, Piwi in echinoids appears to act on a broader set of germ-line regulatory mRNAs than the canonical transposon-only role attributed to the pathway in some model systems, consistent with the emerging view that Piwi proteins participate in mRNA regulation beyond transposon suppression [27]. Small-RNA sequencing of the male gonad of *M. nudus* has identified some 13,000 piRNA-like RNAs, predominantly 28 nt in length, clustered in the genome and matching DNA transposons and retrotransposons, a subset of which was also detected in the ovary [92]; their dependence on sex and gametogenic stage, and their direct targets in the adult gonad, remain unresolved.

Nanos2 belongs to the Nanos family of Zn-finger, RNA-binding proteins that function as translational repressors of specific target mRNAs, typically acting in complex with binding factors on 3′-UTR Nanos Response Elements [93,94]. In the metazoan germ line, Nanos2 is required for the maintenance of an undifferentiated germ-line state and for the specification of germ-cell identity, and its loss produces germ-line depletion in the systems where it has been characterized functionally. In adult echinoids, Nanos2 has emerged as a particularly informative marker of female germ-cell identity. Functional evidence in *S. purpuratus* comes from the translational state of the germ line itself: primordial germ cells synthesize protein at a small fraction of the rate of adjacent somatic cells, and either morpholino knockdown or or clustered regularly interspaced short palindromic repeats/CRISPR-associated protein 9 (CRISPR/Cas9) knockout of *nanos2* partially relieves this quiescence, raising general translation in the germ cells by roughly half [95]. In *M. nudus*, *Mn-nanos2* transcript levels show a pronounced sexually dimorphic and stage-dependent expression pattern. Ovarian transcript abundance rises up to 81-fold above stage I levels during the pre-gametogenic phase of nutritive-phagocyte renewal [28]. In situ hybridization localizes ovarian *Mn-nanos2* expression specifically to female germ cells, while in the testis, expression is detected in both nutritive phagocytes and male germ cells [28].

Functional characterization by RNA interference (RNAi) knockdown of *Mn-nanos2* reduces the expression of *nanos2* itself and of *boule*, a conserved germ-line RNA-binding protein, and coincides with upregulation of *FoxL2* [28,29,79]. This last result establishes a correlation between Nanos2 loss and *FoxL2* derepression in *M. nudus*, though the mechanistic basis of this correlation has not been resolved. Considered together, the *M. nudus* work supports the use of *nanos2* as a stage-specific marker of ovarian germ cells and provides one of the few instances of reverse-genetic functional characterization of a germ-line factor in an adult echinoid gonad. Its stage-locked expression makes it a candidate molecular staging marker for aquaculture, where gametogenic stage is currently assessed almost entirely by histological sectioning, but its germ-cell specificity holds only in the ovary and rests on a single study in one species.

### 3.3. Regulation and Function of Nutritive Phagocytes

If the germinal compartment has yielded the field’s clearest example of a functionally characterized regulator, the somatic compartment that sustains it remains defined largely at the descriptive level, despite biochemical evidence that its principal product operates well beyond passive nutrient storage. The MYP binds zinc, and the coelomic-fluid isoform (CFMYP, ~180 kDa) carries a greater zinc-binding capacity than the egg isoform (EGMYP, ~170 kDa) into which it is partly processed [96]. Because the digestive tract is a principal site of MYP synthesis, this positions the protein as a carrier that transports zinc from the gut to the ovary and testis through the coelomic fluid, part of it processed to the egg-type form, with loss of zinc-binding capacity, upon incorporation into the gonad [71,96]. This transport shows a sexual asymmetry: in *Pseudocentrotus depressus*, both total zinc content and the ratio of coelomic-fluid to egg-type MYP are higher in ovaries than in testes as gametogenesis approaches, and zinc transport is correspondingly more active in females [97]. These observations recast the accumulation-to-mobilization switch introduced in Section 3.1 as a regulated biochemical program rather than a passive filling and depletion of a reserve.

The transcriptional control of this program has begun to be resolved. Profiling of nuclear-receptor expression across nutritive-phagocyte development in *M. nudus* identified a suite of receptor genes whose expression tracks gonadal growth, and recovered a *myp* promoter carrying conserved elements with putative *chicken ovalbumin upstream promoter–transcription factor (COUP-TF)* binding sites [30]. Subsequent work found *myp* and *COUP-TF* transcripts to be positively correlated through gonadal maturation, with binding and transactivation evidence implicating the receptor in *myp* regulation [98]. This is the first candidate transcriptional regulator identified for the dominant gonadal reserve protein, and it locates a nuclear receptor at the point where nutritional and endocrine input might converge on yield. What the histological and biochemical characterization of nutritive phagocytes, including their biosynthesis and storage of MYP, still does not supply are the signals that govern their proliferation, their stage-dependent transition between accumulation and mobilization, or their relationship to the adjacent germinal compartment. Whether the transfer of reserves from nutritive phagocytes to developing gametes is accompanied by molecular signaling between the two compartments, or reflects material transfer alone, has not been established and remains an open question of direct relevance to gonadal physiology. Two kinds of evidence bear on it. Within the somatic compartment, the candidate regulators identified so far act on reserve synthesis itself: *COUP-TF* [98] and, more recently, hepatocyte nuclear factor 4 (HNF4), which localizes to nutritive-phagocyte nuclei in both sexes of *M. nudus* and binds a direct repeat 1 (DR1) element in the *myp* promoter [99]; neither has been linked to a signal originating in the germ line. Between compartments, the only cell-resolved map comes from the sea star ovary, where single-cell data predict ligands and receptors of the Wnt, transforming growth factor-β (TGF-β), Delta/Notch, Hedgehog, and retinoic-acid pathways distributed across somatic and germ-line clusters [84]. These interactions are inferred from expression, have not been tested functionally, and have not been examined in an echinoid. The one soma-to-germ-line signal demonstrated in echinoderms, the relaxin-like gonad-stimulating peptide that acts on asteroid follicle cells to induce 1-methyladenine (Section 5.3), concerns oocyte maturation rather than the accumulation-to-mobilization switch. The gap carries particular weight for aquaculture: because GSI is largely a function of nutritive-phagocyte mass and its stored reserves, the regulation of nutritive-phagocyte accumulation and mobilization is the most proximate molecular determinant of yield. Resolving the signals that time this transition, and the factors that set the balance between reserve storage and gametogenic drawdown, would be a necessary step toward directing gonad yield through defined regulatory targets rather than empirical feed adjustment. Beyond this shared somatic tissue, the factors that differentiate the two gonadal sexes are the subject of the transcriptomic evidence examined next.

## 4. Transcriptomic Signatures of Gonadal Sex Differentiation and Maturation

Of the four mechanistic perspectives integrated in this review, sex-biased gonadal transcriptomics provides the most reproducible body of primary evidence on the molecular state of male and female gonads in commercially relevant echinoids. Most of this evidence comes from gametogenic adult gonads, in which sex-biased expression is dominated by gamete-specific transcripts and by differences in cellular composition (Section 2.4); such expression follows from sex rather than informing how sex is determined. Only data from pre-metamorphic larvae [22] and from juveniles sampled around gonadal differentiation [25] bear on the timing of sex determination and differentiation, and they are identified as such where they are discussed. This section examines the specific factors identified across multiple gonadal transcriptomic studies: the sexually dimorphic transcription factors *Dmrt1* and *FoxL2*, together with associated lipid-biosynthesis and gametogenic gene sets. Throughout, it assesses the extent to which these findings converge across species.

### 4.1. Sexually Dimorphic Transcription Factors: Dmrt1 and FoxL2

Among the sex-determination transcription factors identified across echinoid gonadal transcriptomes, *Dmrt1* and *FoxL2* have received the most direct characterization in sea urchins. In *M. nudus*, *Dmrt1* (*Mn-Dmrt1*) shows testis-specific expression, with transcripts and protein specifically localized to spermatogonia and spermatocytes, identifying it as a candidate male germ-cell marker and a probable regulator of spermatogenic entry [26,64]. Promoter analysis of the 1441 bp 5′-upstream region of *Mn-Dmrt1* identified a positive regulatory element between −1197 and −968 and a negative regulatory element between −1441 and −1198, both containing binding sites for transcription factors including Sp1, Egr1, Sox5, CEBP, GATA, and SRY, indicating that *Dmrt1* in sea urchins is subject to complex upstream regulation by multiple transcription factor families conserved across metazoans [26]. *FoxL2*, previously introduced in Section 3.1 as the marker on which the inferential assignment of the follicle-cell cluster rests, occupies a less settled position, and the available evidence assigns it opposite sex associations depending on the level at which it is measured. At the cell-type level, single-cell profiling of the *S. purpuratus* ovary recovers a *FoxL2*-enriched somatic cluster assigned to follicle cells [65]. At the whole-tissue level, however, *Foxl2* transcript abundance in *M. nudus* is higher in the testis than in the ovary at matched developmental stages [29], and knockdown of *Mn-nanos2* raises ovarian *Foxl2* while driving male gonads to phenocopy the ovarian expression level [28]. We found no report of a functional perturbation of *FoxL2* in any echinoid, so its regulatory relationship to *Dmrt1* in this class remains undetermined. Comparative data supply no default expectation: in the holothuroid *A. japonicus*, *Foxl2* knockdown downregulates *Dmrt1* rather than derepressing it [100], the opposite of the reciprocal derepression predicted by the vertebrate paradigm of mutual *Dmrt1*–*FoxL2* antagonism. The reciprocal experiment in the same species gave a time-dependent result: RNAi knockdown of *Dmrt1* in adult testes lowered *Foxl2* expression at three days and raised it 1.7- to 2.2-fold at seven to ten days, without detectable histological change [101]. Whether that paradigm applies to echinoids at all is therefore open, and an adult knockdown would provide the first direct test.

Beyond *M. nudus*, genes annotated as *Dmrt1* and *FoxL2* have been identified in *L. albus* testis transcriptomes [20] and in *S. purpuratus*, where a case of naturally occurring hermaphroditism identified *Dmrt1* as the first sex-specific marker in the phylum expressed in non-gonadal somatic tissues, which allowed the sexual identity of oral and aboral body tissues to be traced [23]. In the gonad of *M. nudus*, by contrast, *Dmrt1* transcripts and protein localize to spermatogonia and spermatocytes [26], and we found no report, as of the search date, of whether gonadal somatic cells express the gene in either species. The name itself also calls for caution: a genome-wide survey of eleven echinoderms, including *S. purpuratus*, identified no gene orthologous to vertebrate *Dmrt1* and placed the echinoid genes annotated under that name in a supported doublesex-like class [102]. Inferences drawn from the vertebrate role of *Dmrt1* therefore rest on shared DM-domain structure rather than on established orthology. Transcriptomic profiling of the *L. albus* male gonad identified a comprehensive catalog of spermatogenesis-associated genes. This catalog includes forkhead-box family transcription factors (*FoxL2*, FoxX, FoxQ2, FoxN1/4) as well as spermatogenesis-specific kinases (TSSK4, VRK1, HASPIN, MAK), *Dmrt1*, and *rar* (retinoic acid receptor) transcripts. This coverage indicates that the molecular machinery of echinoid spermatogenesis is substantially shared with that of vertebrate gametogenesis, albeit with lineage-specific features that have not yet been functionally characterized.

### 4.2. Evolutionary Conservation of Sex Markers: Transcriptomic Surveys Across Species

Reference gonadal transcriptomes have now been generated for multiple commercially important euechinoids, and their outputs converge on a broadly consistent picture despite differences in species, geography, and methodological details. De novo assembly of paired male and female *P. lividus* gonad transcriptomes yielded 53,865 transcripts (BUSCO completeness > 97%) and a curated catalog of sex-related and lipid-biosynthesis genes framed explicitly as an aquaculture resource [21]. In *L. variegatus*, comparison of ovary and testis transcriptomes identified approximately 1437 genes significantly enriched in ovaries and 1066 in testes, with 101 and 361 transcripts showing ovary- and testis-exclusive expression respectively. A substantial fraction of these early sex-biased transcripts lack annotated identity or recognizable orthologs in public databases [22]. The same study found that meiotic and candidate sex-determination genes are already expressed before metamorphosis, and that individual larvae differ in the extent to which they express male or female gonadal hallmarks, the only data so far that bear directly on when sex determination begins in an echinoid. In *M. nudus*, integrated transcriptomic and metabolomic profiling of differentiated versus undifferentiated gonads, with morphological differentiation at a test diameter of approximately 40 mm, identified differentially expressed genes and metabolites enriched in retinol metabolism, steroid biosynthesis, and amino-acid metabolic pathways, alongside sex-biased expression of components of the transforming growth factor-β (TGF-β) signaling pathway, a signaling system with conserved roles in gonadal differentiation and gametogenesis across animals [25,26]. In *L. albus*, transcriptomic characterization of the male gonad provided functional annotations for over 73% of assembled transcripts, cataloging genes relevant to sexual reproduction, meiosis, sex differentiation, sperm motility, and fertilization [20]. A multi-tissue reference transcriptome that includes juvenile gonads is also available for this species [103], but no sex-comparative or stage-resolved gonadal dataset has been reported for *L. albus*, even though it supports one of the largest echinoid fisheries. The molecular evidence reviewed here therefore applies to this species largely by inference from other euechinoids, a limitation of direct relevance to Chilean aquaculture.

Despite substantial differences in the absolute numbers and functional annotations of sex-biased transcripts across these datasets, several transcription factor families recur consistently as sex-differentiation candidates. Forkhead-box proteins (*FoxL2*-FoxX-FoxQ2 cluster) in female-biased datasets and *Dmrt1* in male-biased datasets emerge as the most broadly conserved sex-determination candidate module across echinoid species examined to date [20,21,26]. Sox-family transcription factors also recur across datasets but with notable cross-species discordance: Sox9 has been identified in *M. nudus* gonadal transcriptomes as a candidate sex-differentiation regulator but was not recovered in the *P. lividus* male-female gonad catalog, suggesting either species-specific expression differences or annotation/assembly artifacts that comparative work has not yet resolved. TGF-β pathway components, including Smads and receptor subunits, likewise show sex-biased expression in *M. nudus*, co-occurring with the differentially expressed metabolites of retinol metabolism [25]. Germ-cell specification and maintenance genes, including *vasa*, *nanos2*, *boule*, and *piwi* homologs, appear across multiple datasets with the sex- and stage-specific expression patterns described in Section 3, confirming that these classical germ-line markers are transcriptionally regulated in a sex- and stage-dependent manner in adult gonads rather than expressed at constitutive basal levels. Across these datasets, the convergences define a shared core of candidate sex-differentiation regulators that is at least partially conserved across the echinoid species surveyed to date. This core spans four regulatory levels: *Dmrt1* and *FoxL2* at the transcription-factor level, TGF-β signaling at the pathway level, retinol and steroid metabolism at the metabolic level, and the germ-line RNA-binding proteins as cell-type markers [20,21,25,26,104].

A striking observation shared across datasets is that a substantial fraction of sex-biased transcripts (30–50% across analyses) are species-specific or lack recognizable functional annotation. This resolves one of the observations flagged in the introduction of this review, namely the sizeable and largely uncharacterized transcript pool that emerges alongside the more familiar sex-differentiation candidates in every gonadal transcriptome examined. The consequence is that a significant portion of echinoid sex-differentiation biology is poorly resolved by comparative-genomic approaches and can only be characterized through direct experimental work within the species of interest. This observation also provides an epistemic anchor for the review’s overall structure: cross-species convergence establishes the shared regulatory core, while species-specific transcripts identify the lineage-specific elaborations that must be resolved on a species-by-species basis to inform aquaculture-oriented applications. The regulatory mechanisms that operate on this core such as the endocrine and retinoic acid signaling systems that presumably link environmental and developmental inputs to the transcriptomic responses characterized above, remain the least resolved layer of the system and are the subject of the sections that follow.

## 5. Endocrine and Neuroendocrine Modulation of Gonad Development and Maturation: Emerging Evidence and Unresolved Paradoxes

Endocrine involvement in echinoid gonad development and gametogenic maturation has been proposed for several decades and, as noted in the introduction, represents the most tentatively supported of the four mechanistic perspectives integrated in this review. Three paradoxes organize this section. First, vertebrate-type sex steroids (estradiol, testosterone, and their intermediates) have been repeatedly detected biochemically in echinoid gonadal tissue, yet the aromatase-encoding gene *cyp19A*, canonically responsible for estrogen biosynthesis from androgen precursors in vertebrates, is absent from non-chordate genomes, including all sequenced echinoid genomes examined to date. Second, the estrogen-related receptor (ERR/NR3B), an orphan NR structurally related to vertebrate estrogen receptors but without a defined endogenous ligand in echinoids, has been characterized in *S. purpuratus* as a candidate reproductive regulator, though its role in gonadal development has not been tested functionally. Third, exogenous steroid supplementation experiments in several echinoid species have produced heterogeneous results across species and protocols; the aggregate literature therefore establishes that gonadal tissue can respond to steroid administration but does not converge on a consistent mechanism or set of active compounds. These paradoxes are examined across Section 5.1 and Section 5.2, beginning with the biosynthetic infrastructure that would need to underpin any downstream endocrine function.

### 5.1. Echinoid Steroidogenesis and Cholesterol Regulation

One of the most persistent and consequential unresolved questions in echinoid reproductive endocrinology is whether echinoderms can synthesize sex steroids endogenously or whether the vertebrate-type steroids repeatedly detected in their gonadal tissue originate from environmental uptake. Vertebrate-type sex steroids, including estradiol and testosterone, were first reported in sea urchin tissue over 60 years ago and have been confirmed more recently by immunoassay and mass-spectrometry-based metabolomics in *M. nudus* gonads and in *P. lividus* gonadal tissue across reproductive stages [105,106]. Yet comprehensive phylogenomic surveys fail to consistently identify any chordate-type aromatase (*cyp19A*) or classical vertebrate-type steroid receptors (estrogen, androgen and progesterone receptors; ER, AR, PR) in any non-chordate genome; evolutionary analyses of cytochrome P450 hydroxylases indicate that the steroidogenic CYP11-CYP17-CYP19 pathway evolved sequentially within chordates, with CYP19 being the most phylogenetically restricted, present only in chordates and absent in echinoderms, hemichordates, and all protostome lineages [34]. Estrogen biosynthesis via the vertebrate-type aromatase reaction therefore appears unavailable in echinoids at the genomic level, given the absence of an identified *cyp19A* ortholog; whether estrogens are produced by alternative routes remains unresolved.

This discrepancy between steroid biochemistry and genomic evidence admits several non-exclusive explanations. One is that echinoderms possess a non-canonical aromatization route, mediated by a paralogous or convergently evolved CYP enzyme; CYP11-like sequences have been identified in *S. purpuratus* and CYP11A activity has been reported in echinoid tissue, suggesting that upstream steroidogenic capacity may be present even if a canonical CYP19 aromatization step is not [34]. Expression evidence from gonadal tissue is consistent with this possibility: characterization of the cytochrome P450 complement in *M. nudus* gonads identified sexually dimorphic CYP transcripts, among them a *CYP17A1*-like gene whose expression accompanies testicular maturation [107]. Expression of a candidate enzyme is not activity, so the finding locates the question in the relevant tissue without resolving it. A second is that steroids detected in gonadal tissue derive substantially from passive uptake from seawater or dietary sources, a mechanism documented in marine invertebrates exposed to steroid-rich coastal water and endocrine-disrupting compounds [37,106,108]. Both explanations are consistent with the available data, and distinguishing between them requires biochemical approaches that have not yet been applied to euechinoids, such as radiolabeled-precursor tracing and heterologous expression of candidate CYP enzymes.

A parallel paradox emerges at the receptor level. Although classical vertebrate-type steroid receptors (ER, AR, PR) are absent from echinoderm genomes, a systematic phylogenomic survey of the *S. purpuratus* genome identified a substantial complement of nuclear-receptor genes representing most metazoan NR families [109], and gonadal transcriptomic profiling in *M. nudus* has since demonstrated that 23 of these receptors are expressed in the gonad during early gametogenesis, 20 of them in both sexes, with four showing significant upregulation during gonadal growth in both sexes, *eip78c* (NR1E), *trβ* (NR1A), *bar* (NR1, group unassigned), *rxrγ* (NR2, group unassigned), and a fifth, *ftz-f1β* (NR5A), in males only [110,111]. Downstream transcriptional infrastructure is also present, although it has been characterized only in embryos: a p160-family steroid-receptor coactivator isolated from *Strongylocentrotus nudus* (now *M. nudus*) interacts with several nuclear receptors, including the sea urchin ERR, and its morpholino knockdown disrupts gastrulation [112]. We found no report, as of the search date, of whether the same coactivator operates in the adult gonad. Within this receptor complement, the estrogen-related receptor (ERR/NR3B), an orphan nuclear receptor of the NR3 family structurally related to vertebrate estrogen receptors, has drawn particular attention as a candidate reproductive regulator. In *M. nudus*, *err* transcripts were detected only in the testis at the onset of gametogenesis, and their abundance increased in males and decreased in females between the recovery and growth stages [110]. In vertebrates, ERR functions as a metabolic and mitochondrial-biogenesis regulator responsive to endogenous fatty-acid and cholesterol-derived ligands rather than to estrogen itself, and comparable functional conservation has been documented in a urochordate deuterostome [113]. Extending this ligand-independent metabolic role to the echinoid receptor requires inference from vertebrates through urochordates to echinoderms and should be read as correspondingly provisional. In echinoids, however, the endogenous ligand remains undefined, and no perturbation study has established whether echinoid ERR mediates gonadal responses to steroids, metabolic intermediates, or environmental cues. The receptor thus exists as a plausible transducer of endocrine or metabolic signals within the gonad, without confirmed function in gonadal development or gametogenic maturation. One interpretive route through this receptor-level paradox invokes non-genomic (membrane-associated) steroid signaling as an alternative to classical nuclear-receptor mediation [114], a hypothesis that will be revisited in light of the exogenous-supplementation evidence developed in Section 5.2.

### 5.2. Steroid Effects on Gametogenesis: Functional Evidence from Exogenous Supplementation

Endogenous vertebrate-type steroids have been quantified across the gametogenic cycle in multiple echinoid species. In *L. variegatus*, testosterone and estradiol both rose during gonadal growth and dropped after spawning [115,116]. In *P. lividus*, testosterone varied significantly across the male reproductive cycle and estradiol was consistently lower in males than females, though neither analyte correlated cleanly with gonad index [105]; liquid chromatography–mass spectrometry (LC–MS) quantification of endogenous estradiol subsequently confirmed its presence at physiological levels [117]. Against this baseline of measurable endogenous steroids, several groups have since tested whether dietary or waterborne administration of exogenous steroids modulates gametogenic outcomes. In *P. depressus*, a 30-day dietary supplementation with androstenedione or estrone significantly raised the male gonad index in juvenile animals, with estrone additionally promoting spermatogenesis histologically; estradiol-17β produced no significant effect under the same protocol [118]. In *L. variegatus*, 36-day dietary administration of estradiol, progesterone and testosterone produced steroid- and sex-specific effects: 5α-reduced progestins (progesterone metabolites) promoted nutritive-phagocyte reserve accumulation, stimulated testis growth, and inhibited spermatogenic column formation; estradiol raised the mean ovary index by 54% relative to control but simultaneously reduced oocyte diameter; testosterone promoted oocyte growth without raising the ovary index [116]. Inhibition of 5α-reductase by finasteride in the same species had earlier confirmed that the 5α-reduction step is active in both ovary and testis [119]. The *P. depressus* and *L. variegatus* dietary results are the two clearest positive findings in the field, suggesting that echinoid gonadal tissue can respond to exogenous steroid administration with measurable histological and morphometric outcomes.

Against these positive findings, a distinct body of work in *P. lividus* has repeatedly returned null results for natural sex steroids. Estradiol delivered by peristomial injection at three concentrations over two- and twelve-week periods induced no marked variation in reproductive parameters, and gonad index tracked feed intake more closely than estradiol treatment [120]. Combined in vivo dietary and in vitro ovarian cell-culture exposure to estradiol and testosterone likewise failed to influence gonad and gamete growth or MYP content [117]. For estradiol specifically, these null results agree with the *P. depressus* study, in which estradiol-17β was also without effect [118]; the divergence for this steroid lies between these two species and *L. variegatus* and *S. intermedius*, in which estradiol altered ovary index or sex-related gene expression [8,115,116]. The studies also differ in the steroid tested, the life stage and gonadal stage of the animals, and the route, dose and duration of exposure (Table 1), so the extent to which these contrasts reflect differences between species rather than between protocols cannot yet be determined. One interpretive framework proposed to account for this lack of response invokes non-genomic (membrane-associated) steroid signaling rather than classical nuclear-receptor mediation [114], a hypothesis for which we found no experimental test in an echinoid. A candidate mediator is nonetheless annotated: the *S. purpuratus* genome contains a membrane-associated progesterone receptor component 1 ortholog (*Sp-Pgrmc1*, *LOC578050*) carrying an intact cytochrome b5-like heme/steroid-binding domain, for which no functional study of this gene in an echinoid was found [121]. Its presence supplies a specific molecular target for testing whether the responses reviewed here are transduced at the membrane rather than through nuclear receptors. *P. lividus* gonadal tissue is not universally unresponsive to steroid-pathway compounds. Waterborne exposure to the synthetic estrogen ethinylestradiol (EE2) at environmentally relevant concentrations produced sex-specific effects: females exposed to 10 ng/L EE2 for 28 days showed increased gonadal maturation, while males exposed to EE2 exhibited increased frequencies of low-development gonad stages [122]. Triphenyltin exposure altered *P. lividus* androgen metabolism (increased 5α-reductase activity and testosterone-conjugating activity) and modulated spermatogenesis in a concentration-dependent manner [123]. The species therefore responds functionally to synthetic estrogens and steroid-metabolism-disrupting compounds while showing no detectable response to natural sex steroids under the doses, routes and durations tested so far, a pattern that constrains but does not resolve the mechanistic interpretation.

Molecular characterization of the transcriptional response to exogenous steroid administration is limited to a single study. In *S. intermedius*, 58 days of dietary estradiol supplementation altered the expression of conserved testis-differentiation, ovary-differentiation, and estrogen-metabolism genes in a stage-dependent manner [8]. We found no other transcript-level response to exogenous steroids reported for an echinoid. Taken with the histological effects reported in *P. depressus* and *L. variegatus*, this result suggests that at least some echinoid species mount a molecular gonadal response to exogenous steroids. Viewed across species, however, the pattern of effects is heterogeneous: dietary androstenedione and estrone stimulated male gonad growth in *P. depressus*; dietary 5α-reduced progestins reorganized somatic and germinal compartments in *L. variegatus*; dietary estradiol modulated sex-differentiation gene expression in *S. intermedius*; and dietary, injected, and in vitro estradiol failed to affect gametogenesis in *P. lividus*. Because no two of these studies share the same steroid, life stage, route, dose and duration, this heterogeneity cannot yet be attributed to species differences. The receptors mediating the observed effects in any of the responsive species have not been identified.

Several gaps in the current evidence base limit how much interpretive weight this literature can bear. Of the commercially most important aquaculture species, only *S. intermedius* has been characterized at the transcript level; *M. nudus*, *M. franciscanus*, *L. albus*, and *S. droebachiensis* have not been tested at the exogenous-supplementation level with gonadal endpoints. A natural extension of the aquaculture-formulation logic that dominates the field would be to supplement steroidogenic precursors and measure gonadal-endocrine endpoints, yet we found no report of such an experiment. Proteomic and metabolomic responses to exogenous steroid exposure in gonadal tissue remain unreported to date. The synthesis these functional studies support is correspondingly limited: echinoid gonadal tissue is responsive to some exogenous steroid administrations in some species under some protocols, without a demonstrated mediating receptor, without a consistent cross-species pattern, and without the molecular resolution that would allow the responses to be interpreted mechanistically.

### 5.3. Neuroendocrine Control of Maturation and Spawning

The steroid and nuclear-receptor systems examined above operate alongside a peptidergic axis that has been characterized mainly outside the Echinoidea. In starfish, the relaxin-like gonad-stimulating peptide (RGP) is a two-chain peptide of the insulin/relaxin superfamily, released from the radial nerve. It has been reported to act on ovarian follicle cells to elicit 1-methyladenine—a purine derivative that serves as the maturation-inducing hormone in asteroids—which is in turn associated with oocyte maturation and spawning [124,125]. A synthetic RGP has also been shown to induce oocyte maturation and ex vivo spawning in a holothurian, though in that class the downstream maturation-inducing hormone does not appear to be 1-methyladenine [126]. Together these findings suggest an ancestral echinoderm role for RGP in reproductive control, with class-specific downstream chemistry. Beyond RGP, starfish also possess gonadotropin-releasing hormone (GnRH) -type and corazonin-type signaling systems, each with an identified ligand–receptor pair, extending the reproductive-neuropeptide repertoire of echinoderms toward the systems familiar from vertebrate reproductive endocrinology [127].

In echinoids, the molecular substrate for such an axis is present: peptidomic and genomic analyses of *S. purpuratus* have described a substantial neuropeptide complement, including 23 confirmed prohormones and a range of peptides recovered from the radial nerves [128,129]. Its function, however, remains largely open. No RGP ortholog has yet been shown to control gonadal maturation in a sea urchin, and the maturation-inducing signal acting downstream of neural input in the echinoid gonad has not been identified. Comparative work in other echinoderms offers a further indication of what such an axis might govern: in gravid starfish, reproductive neuropeptides are expressed in a sex-specific manner and have been linked to the coordination of aggregation and synchronized spawning [130]. This gap is relevant to aquaculture because comparative work indicates what a resolved pathway might afford: peptide-triggered, more synchronized induction of maturation and spawning, which would bear directly on controlled seed production. Establishing whether an echinoid RGP–maturation axis operates is among the more tractable questions the neuroendocrine literature raises.

Across both its steroidal and peptidergic dimensions, the endocrine regulation of the echinoid gonad is defined more by identified components than by demonstrated function. The same characterization extends, in a different form, to the retinoid signaling developed in the following section.

## 6. Retinoic Acid Signaling in Echinoids: From Metamorphosis and Gonadogenesis to Reproduction

Retinoic acid (RA) signaling constitutes the fourth mechanistic perspective integrated in this review and, alongside endocrine signaling developed in Section 5, represents the most tentatively supported of the four lines in the specific context of echinoid gonadal biology. While RA is a well-characterized developmental morphogen in vertebrates, and while its biosynthetic and receptor machinery has been documented across echinoderms at the genomic level, direct functional evidence for RA signaling in the adult gonad of any echinoid species is essentially absent. This section develops the RA line across three subsections. Section 6.1 establishes what RA signaling machinery is present and what is missing at the genomic level in echinoderms, focusing on the receptor complement, the biosynthetic and catabolic enzymes, and the ligand-binding proteins. Section 6.2 reviews functional evidence for RA activity across the echinoderm life cycle, where the strongest data come from metamorphosis studies in non-echinoid echinoderms (starfish, feather stars) rather than from sea urchins themselves. Section 6.3 addresses the specific question of RA involvement in echinoid gonadogenesis.

### 6.1. Conservation and Molecular Machinery of RA Signaling

The RA signaling pathway in vertebrates comprises three functional modules: RA synthesis from dietary retinol via retinol dehydrogenases (RDHs) and aldehyde dehydrogenases (ALDH1A/RALDH); RA transport to nuclear compartments by cellular retinoic-acid-binding proteins (CRABPs); and RA-dependent transcriptional regulation via the RAR–RXR nuclear-receptor heterodimer, with signal attenuation through CYP26-mediated catabolism [36]. Comparative genomic surveys of echinoderms have documented that this machinery is partially conserved, but with notable losses and unresolved biochemistry.

RA catabolism is the most fully conserved module of the pathway in echinoderms. A single *cyp26* gene has been identified in each echinoderm class examined, with functional-domain residues conserved between the echinoderm and human orthologs [36]. An RA-response element (RARE) carrying a two-nucleotide spacer, corresponding to a DR2 configuration, has been identified at 2503 bp upstream of the *S. purpuratus cyp26* gene, and a DR5-type RARE at 3770 bp upstream of the *S. purpuratus hox1* gene [131,132], providing genomic evidence that RA-dependent transcriptional regulation is at least architecturally possible in echinoids. Whether the RAR–RXR heterodimer specifically recognizes these RAREs in vivo remains untested. Because the biosynthetic route to RA remains biochemically undemonstrated in echinoderms, CYP26 provides the most tractable functional readout of an active retinoid response in this group.

The receptor module is likewise present, though its functional properties are only partly resolved. The echinoderm complement consists of a single *rar* and a single *rxr* gene per genome, in contrast with the triplication into *rarα/β/γ* and *rxrα/β/γ* that occurred in the vertebrate lineage [36,133]. Both receptors have been identified in all echinoderm classes examined at the transcriptome or genome level, including *S. purpuratus*. Whether the echinoderm RAR–RXR heterodimer regulates downstream gene expression as it does in vertebrates has not, however, been experimentally demonstrated [36]. Ligand-binding has been characterized only outside the echinoderms: the ancestral RAR of the annelid *Platynereis dumerilii* and the priapulid *Priapulus caudatus* binds RA with low affinity [134,135]. Whether the echinoderm RAR selectively binds all-*trans*-RA or preferentially engages other isomers remains unresolved.

The biosynthetic module, by contrast, shows the most consequential taxonomic gap. Vertebrate RA is synthesized primarily by ALDH1A enzymes (RALDH1/2/3), and *aldh1a* orthologs have been retained across most bilaterian lineages. In echinoderms, orthologs of the ALDH1A subfamily are present in the starfish *Patiria pectinifera*, the feather star *Metacrinus rotundus*, and the brittle star *Amphiura filiformis*, but are absent from the sea urchin *S. purpuratus* and the sea cucumber *Apostichopus japonicus*, a pattern consistent with loss of *aldh1a* in the common ancestor of the echinozoan lineage [36,136,137]. An alternative aldehyde dehydrogenase, *aldh8a1*, has been identified in echinoids and other echinoderms, and was initially proposed as a candidate RA-synthesizing enzyme in taxa lacking *aldh1a*; however, recent biochemical reassignment of vertebrate ALDH8A1 to the kynurenine pathway of tryptophan catabolism [138] has cast doubt on whether *aldh8a1* orthologs support RA biosynthesis in echinozoans. The consequence is that a biochemically demonstrated pathway from retinol to all-*trans*-RA within sea urchin tissue has not been established.

The RA transport module is the least conserved of the four. CRABPs, which deliver RA to nuclear RARs in vertebrates, appear to be a vertebrate-specific innovation; echinoderms possess fatty acid binding protein (FABP)-like genes without clear orthology to vertebrate CRABPs [36]. Whether echinoderm FABP-like proteins fulfill an equivalent transport function, or whether RA is delivered to nuclear receptors by an alternative mechanism, has not been determined. This gap has particular consequences for the non-canonical RA signaling pathway, in which vertebrate FABP5 transports RA to peroxisome proliferator-activated receptor (PPAR)–RXR heterodimers as an alternative to CRABP-mediated delivery to RAR–RXR [139]; whether an equivalent non-canonical route operates in echinoderms cannot presently be established from the available data.

The genomic evidence therefore establishes that echinoids possess the transcriptional and catabolic components of RA signaling (RAR, RXR, CYP26, RAREs) but lack a demonstrated biochemical route from retinol to RA and a transport module orthologous to vertebrate CRABPs. The signaling pathway is thus architecturally present but biochemically incomplete in the components most upstream and downstream of receptor engagement.

### 6.2. Functional Roles of RA Signaling Across the Echinoderm Life Cycle

The functional role of RA signaling in echinoderm development has been established most directly in metamorphosis studies conducted in starfish and feather stars. In the starfish *P. pectinifera*, exogenous RA treatment of competent larvae induced metamorphosis, while pharmacological inhibition of RA synthesis or of RAR-mediated transcription suppressed metamorphosis; the RA-pathway components *aldh1a*, *rar*, and *rxr* were spatially expressed in larval tissues during the pre-settlement period, in an expression pattern consistent with an endogenous role in metamorphic competence and initiation [140]. This finding was subsequently extended to a second starfish species with a different larval type, the lecithotrophic *Astropecten latespinosus*, where exogenous RA likewise induced metamorphosis [141,142]. Conservation across echinoderm classes was then established in the feather star *Antedon serrata*, where exogenous RA induced metamorphosis of doliolaria larvae and inhibition of RA synthesis suppressed it [143]. The demonstrated data therefore span two echinoderm classes—Asteroidea and Crinoidea—and support the interpretation of RA signaling as an ancestral echinoderm regulator of life-history transitions [36].

In sea urchins specifically, the evidence for RA signaling in metamorphosis is less developed. Larval competence and settlement in echinoids have been characterized as regulated by thyroid hormone, histamine, and nitric oxide signaling [144], and a role for RA signaling in sea urchin metamorphosis has not been reported [36]. Direct evidence for RA responsiveness in echinoid cells is nonetheless available at the developmental level. Exposure of *P. lividus* embryos to exogenous all-*trans* RA delayed developmental progression without producing a specific morphological phenotype [145], and treatment of micromere-derived cells from *H. pulcherrimus* embryos with all-*trans* RA induced pseudopodial cable growth [146]. Morpholino knockdown of *rxr* in *M. nudus* embryos produced abnormal early development and complete arrest at the gastrula stage, demonstrating that the RXR receptor is functionally required for embryonic patterning in an echinoid [133]. Read together, these findings establish that echinoid cells and embryos respond to RA and depend on RXR function, though the specific role of RA in echinoid life-history transitions remains formally undemonstrated.

### 6.3. Direct Evidence of Retinoid Signaling in Adult Echinoid Gonads: Gaps in Evidence

The extension of the RA signaling framework from developmental to adult-gonadal contexts in echinoids remains, as of this writing, essentially untested at the functional level. We found no published study in which exogenous retinoic acid was administered to adult sea urchins and gonadal outcomes were measured. No receptor-level perturbation (RAR or RXR knockdown, pharmacological antagonism, or RA-synthesis inhibition) has been conducted in adult echinoid gonadal tissue. As developed in Section 6.2, exogenous RA administration in echinoids has been characterized only in embryonic and isolated cellular systems, and RXR is functionally required for embryonic patterning in *M. nudus*; none of these findings extends to adult gonadal function. What can therefore be stated about RA signaling in adult echinoid gonads is limited to correlative and descriptive evidence at the transcript, receptor-repertoire, and metabolite-abundance levels, alongside cross-taxon comparative inferences from other adult echinoderm tissues.

Two recent studies in *M. nudus* provide the strongest direct signals available. Comprehensive nuclear-receptor expression profiling during early gametogenesis identified 23 nuclear-receptor genes expressed in the gonads, of which four were significantly upregulated during gonadal growth in both sexes [110]. The same study reports two transcripts annotated *rxrα* and *rxrγ*. The primer sequences reported in [110] were mapped in silico to the *S. purpuratus* RefSeq transcript set, and the predicted proteins of the matching loci were aligned with human RXRα (NP_002948; pairwise local alignment). The *rxrα* and *rxrγ* primers matched (0–2 mismatches per primer) two distinct loci (*LOC579018*, 83% amino-acid identity; *LOC579226*, 36% amino-acid identity), of which only one encodes a protein closely similar to vertebrate RXRs. The canonical *rxr* (NR2B), consistent with the single *rxr* gene identified in echinoderm genomes [36,133], therefore corresponds to *rxrα*, which increased in males during gonadal growth, whereas the *rxrγ* transcript upregulated in both sexes appears to represent a divergent NR2 receptor whose group remains to be assigned phylogenetically. Both findings are associational and do not identify a ligand or a downstream transcriptional target. In parallel, integrated transcriptomic and metabolomic analysis of gonadal differentiation identified RA, linoleic acid, and arachidonic acid among the differentially abundant metabolites during gonadal maturation, with retinol metabolism enriched among the differentially expressed pathways and *CYP26*, *CYP1A*, and *CYP3A* showing sex-biased expression [25]. The semi-quantitative detection of retinoic acid in gonadal tissue by untargeted metabolomics is consistent with endogenous RA presence, but no dedicated LC–MS/MS quantification of retinol, retinal, or RA isomers has been performed in echinoid gonadal tissue, and the identities and stoichiometry of the retinoids detected remain to be established. A third piece of adult-gonad evidence concerns receptor heterodimer partnering rather than RA signaling proper: in *P. lividus*, PPAR and RXR were cloned from adult tissue, and the reconstituted PPAR/RXR heterodimer showed concentration-dependent transactivation modulated by tributyltin exposure, with in vivo treatment altering gonadal fatty-acid composition and upregulating *acsl*, *ppar*, and *rxr* in the gonad [147]. This finding establishes that at least one RXR-partnering heterodimer is functionally operational in an adult echinoid gonad, though the ligand context is fatty-acid/lipid rather than retinoid.

Cross-taxon comparative evidence from adult echinoderm tissues extends this correlative base but does not substitute for direct evidence in echinoid gonads. In the sea cucumber *Holothuria glaberrima*, three RAR isoforms and two RXR isoforms have been characterized in adult tissues including the gonad, and pharmacological perturbation of RA signaling—synthesis inhibition with citral, RAR antagonism with LE135, agonism with tazarotene—reduced rudiment size and modulated cell proliferation and dedifferentiation during intestinal regeneration [148,149]. This is currently the closest available functional demonstration of RA-signaling activity in any adult echinoderm tissue, though the demonstrated context is regeneration rather than gonadal function. Functional RA signaling in echinoderm metamorphosis has been established in the starfish *Patiria pectinifera* [140] and the feather star *Antedon serrata* [143], as developed in Section 6.2. For the class Ophiuroidea, no functional RA study was found: RA-machinery gene presence in *Amphiura filiformis* is documented at the genomic level only [36]. The available cross-taxon evidence therefore licenses the inference that echinoderms possess a functionally RA-responsive apparatus but does not demonstrate a specific gonadal role in any echinoid.

The identification of retinoids in echinoid gonadal tissue raises the question of their biochemical origin. In vertebrates, retinoic acid is synthesized from dietary retinol via retinol dehydrogenases and ALDH1A-family aldehyde dehydrogenases. In echinozoans this canonical route is compromised: ALDH1A orthologs are absent from *S. purpuratus* and *A. japonicus*, consistent with loss in the echinozoan common ancestor, as developed in Section 6.1. This unresolved biosynthetic route has motivated attention to an alternative ligand-provisioning framework centered on direct enzymatic cleavage of dietary carotenoids to retinoids, bypassing the requirement for aldehyde-dehydrogenase-mediated synthesis from retinol. The relevant biochemical background is independently established: sea urchin gonads accumulate carotenoids at high concentrations, with echinenone reported as the dominant gonadal carotenoid in commercially exploited euechinoids, where it accounts for approximately 50–60% of the total pigment and where the 9′-*cis* form is present at roughly 10-fold the concentration of the all-*trans* form [85]. Figure 3 traces this proposed axis from dietary β-carotene to the retinoid receptors, indicating the evidence status of each step. Comparable dominance of echinenone in the mature gonad has been documented across additional commercially relevant echinoid species, including *P. depressus*, *Anthocidaris crassispina*, and *Psammechinus miliaris* [86,150,151,152]. The bioconversion of dietary β-carotene to echinenone occurs primarily in the gut wall, from which the product is subsequently incorporated into gonadal tissue [153,154]. How carotenoids are then delivered to and retained within the gonad is less clear, and current evidence points to two complementary mechanisms. Direct carrier binding is supported by a fatty acid-binding protein (FABP)-like echinenone-binding protein (EBP) isolated from *Evechinus chloroticus*, which binds echinenone and remains the only biochemically characterized carotenoid carrier in an echinoid gonad [155]. A lipoprotein-mediated route is suggested by transcriptomic work in *S. intermedius*, where superior gonad coloration tracks with upregulation of the low-density lipoprotein (LDL) assembly and uptake machinery—*apoB-100*, *the LDL receptor (LDLR*), and scavenger receptor *class B type 1 (SR-B1*)—paralleling the receptor-mediated uptake of LDL–carotenoid complexes described in other animals [24]. Neither route has yet been confirmed by direct measurement of carotenoid flux in echinoid gonadal tissue.

**Figure 3:**
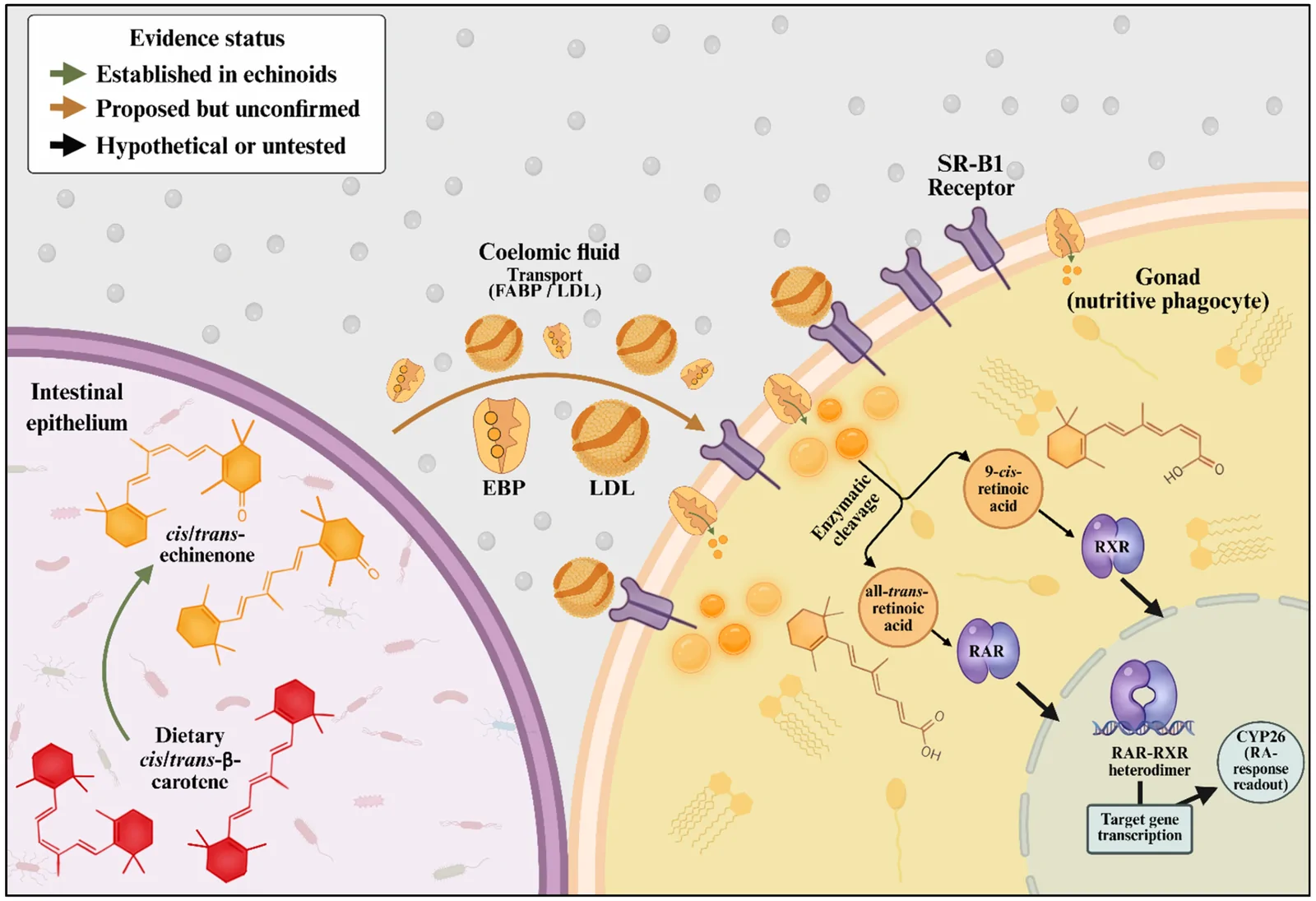
Proposed carotenoid-to-retinoid signaling axis in the euechinoid gonad, with evidence status by step. The pathway is shown across three anatomical zones. In the intestinal epithelium, dietary *cis*/*trans*-β-carotene is converted to *cis*/*trans*-echinenone [153,154]. Echinenone is then transported across the coelomic fluid to the gonad, a step proposed to involve a FABP-like echinenone-binding protein and a lipoprotein route through SR-B1/LDL-receptor-mediated uptake [24,155]. Within the nutritive phagocyte, echinenone accumulates in lipid droplets, where the 9′-*cis* isomer predominates [85]. Enzymatic cleavage of this pool to retinoid ligands, with 9-*cis*-retinoic acid as the hypothetical product of 9′-*cis*-echinenone cleavage, has not been demonstrated in any echinoid. The proposed downstream chain would converge on the RAR–RXR heterodimer, driving target-gene transcription with CYP26 as the RA-response readout. Colored-arrow pathways denote the evidence status of each step: green, established in echinoids; amber, proposed but unconfirmed; black, hypothetical or untested. Abbreviations: FABP, fatty acid binding protein; LDL, low-density lipoprotein; SR-B1, scavenger receptor class B type 1. Created in BioRender. Rodríguez, P. (2026) https://BioRender.com/miibhxm (accessed on 25 September 2026).

The critical linking step in the carotenoid-ligand framework is the enzymatic cleavage of gonadal carotenoids to yield retinoid ligands available for nuclear-receptor activation. Carotenoid oxygenases (β-carotene oxygenases; BCO1, catalyzing central 15,15′ cleavage of β-carotene to retinal, and BCO2, catalyzing eccentric 9′,10′ cleavage) belong to a superfamily broadly distributed across metazoans, which includes an ancestral BCO-like assemblage recovered outside the vertebrate BCO1/BCO2 pair [156]. Functional characterization in invertebrates has been achieved in molluscs: RNA interference against BCO2 in the Yesso scallop *Patinopecten yessoensis* altered carotenoid deposition and identified a BCO-like paralog as causal for the pigmented phenotype [157]. The echinoid complement is minimal and unresolved. A single carotenoid oxygenase superfamily member is annotated in the *S. purpuratus* genome (*LOC752114*), listed provisionally under both *rpe65* and β-carotene 15,15′-monooxygenase (*Sp-Bcmo1*) synonyms and without annotated paralogs [121], mirroring the single-copy condition of *rar* and *rxr* described in Section 6.1. Because these enzymes share the RPE65 domain, and because functional RPE65 orthologs have been reported only in vertebrates, the assignment between an isomerohydrolase and a carotenoid-cleavage function cannot be made from current annotation alone. We found no functional study of this gene, no characterized carotenoid cleavage oxygenase in any echinoid, no carotenoid-to-retinoid conversion assay in gonadal tissue, and no endogenous retinoid attributed to a carotenoid-cleavage step. The biochemical continuity from gonadal carotenoid pool to nuclear-receptor-activating retinoid ligand thus remains a plausible but untested hypothesis in this taxon.

The overall interpretive weight that can be placed on RA signaling as a functional mechanism in adult echinoid gonadal biology is therefore constrained by an evidence base in which every functional criterion—exogenous RA administration, receptor perturbation, retinoid quantification, RA-synthesizing enzymatic route, and functional demonstration of carotenoid-to-retinoid conversion—is currently untested at the adult gonad level. The two documented adult-gonad signals in *M. nudus* [25,110] and the *P. lividus* PPAR/RXR finding [147] collectively establish that RA-pathway components are expressed and dynamically regulated in adult echinoid gonads, that RA-related metabolites are present, and that at least one RXR-partnering heterodimer is functionally operational. These signals justify treating RA signaling as a candidate regulatory axis in adult echinoid gonadal biology worthy of experimental investigation, but they do not, individually or collectively, demonstrate a functional role.

Establishing this pathway experimentally would carry direct implications for aquaculture. If dietary carotenoids can be shown to yield receptor-active retinoids that modulate gonadal maturation, carotenoid formulation would acquire a second function. Beyond its established commercial role in roe pigmentation, it would become a potential lever on the reproductive and developmental program itself. Whether such a lever exists remains contingent on the functional demonstrations catalogued above, none of which has yet been undertaken in an echinoid.

Carotenoids are not the only pigment class of echinoids. Echinoids also utilize polyhydroxylated naphthoquinones, including echinochrome A and the spinochromes, through an endogenous polyketide pathway; knockout of the polyketide synthase gene *pks1* abolishes pigmentation at every life stage [158]. These pigments have been quantified in eggs and embryos [159] and are present in the gonad, although at the lowest concentrations of the body compartments examined [160]. Gonad color, by contrast, is attributed to carotenoids, principally echinenone [85], and we found no study relating quinoid content to gonad color or commercial quality; for this reason, the quinoid pigments were not treated among the levers on gonadal outcome. We likewise found no report, as of the search date, of an interaction between quinoid pigments and retinoid synthesis, transport, or receptor signaling in echinoderms. Whether naphthoquinones deposited in the oocyte influence gonadal retinoid status, or share an antioxidant function with carotenoids during gametogenesis, remains to be established.

## 7. Levers on Gonadal Outcome: From Molecular Control Points to Aquaculture Targets

The preceding sections characterized the molecular biology of the echinoid gonad along four largely independent lines. Reorganized around several outcomes of interest to aquaculture, that same body of knowledge can be read as a set of mechanistic control points, each relevant to a distinct production objective: the conditioning of broodstock, the accumulation of gonadal reserves associated with yield, the pigmentation associated with roe quality, and the developmental program underlying seed production. These are not the only objectives an aquaculture program might pursue, but they are among those for which the molecular literature reviewed here offers some mechanistic traction. Table 2 maps each objective to the mechanisms that appear to govern it, the species in which those mechanisms have been examined, the current state of knowledge, and the control lever that mechanism might afford.

The mapping is uneven in confidence: some levers are supported by well-characterized biology, whereas others remain contingent on demonstrations the field has not yet produced. A further limit applies to every lever in Table 2. Nearly all cell-level evidence comes from *S. purpuratus* and *L. variegatus*, whereas the species of greatest aquaculture interest, *M. nudus*, *L. albus*, *P. lividus*, and *S. intermedius*, contribute tissue-level transcriptomes, histology, and feeding trials; of these, only *M. nudus* and *S. intermedius* belong to the same family as *S. purpuratus*. Cross-species alignment of ovarian single-cell atlases indicates that cell states do not transfer uniformly: between sea star and sea urchin ovaries, oocyte and muscle clusters aligned consistently, whereas follicle-cell clusters aligned too heterogeneously to support confident identity assignment [84]. We found no single-cell atlas of the gonad of any primary aquaculture species as of the search date, so the transfer of somatic cell states from model species to these species remains an assumption to be tested rather than a basis the levers proposed here can take for granted.

Cutting across these objectives is the environmental regime under which the animals are held. Temperature and photoperiod entrain the gonadal cycle and are already manipulated at production scale: controlled-temperature holding improves gonad quality in barren-collected animals [14], compressed photoperiod cycles advance maturation in *S. intermedius* [161], and combined photothermal regimes shift gonadal stage distribution in *P. lividus* [162], while sustained warming reshapes the gonadal metabolome in ways that reach the sensory traits determining market value [163]. These responses are established at the phenotypic level alone. We found no study connecting an identified photoreceptive or thermosensory pathway to the transcriptional state of the adult gonad, so the most widely applied lever in echinoid aquaculture operates without a mechanistic account of how it acts.

A second cross-cutting input is microbial. Dietary probiotic supplementation improves gonad development and quality in cultured echinoids [164], and *Bacillus* supplementation of a plant-meal diet improved survival and gonad coloration in *S. purpuratus* [4]. Two distinct mechanisms are conflated in this literature and warrant separation. The first is modulation of host physiology through the gut microbiome, whereby bacterial colonization alters digestion, nutrient assimilation, or immune status. The second is direct enzymatic bioactivation of dietary substrates, in which bacterial glycoside hydrolases liberate aglycones from plant-derived glucosides before or during absorption. Only the second predicts a defined molecular route from feed composition to the signaling systems reviewed above, and it has not been demonstrated in any echinoid: we found no measurement of bacterial enzyme activity toward plant-derived substrates in sea urchin gut, and no quantification of aglycone in gonadal tissue. The lever proposed in Table 2 therefore rests on a mechanism that remains to be established and is offered as prospective on that basis.

Read across the four objectives, the levers differ less in plausibility than in readiness. Two are grounded in reasonably well-characterized biology. Marker-guided conditioning of broodstock would draw on candidate stage markers such as *nanos2* and *Dmrt1* to inform the timing of established photothermal and dietary regimes, and the dietary loading of nutritive-phagocyte reserves offers mechanistic framing for a practice the industry already applies empirically. A third lever, dietary carotenoid supplementation for pigmentation, is well established at the level of outcome, even though the physiological link between pigment intake and gonadal maturation remains open. The remaining possibilities are more prospective. The enzymatic bioactivation of dietary substrates, the carotenoid-to-retinoid conversion that might connect pigment metabolism to maturation, and the manipulation of early developmental signaling each describe a point at which intervention could become feasible if the underlying mechanism were resolved, rather than one that is actionable at present. The value of the mapping lies in this distinction: it indicates which production outcomes the current molecular understanding can begin to inform, and which await the more targeted experimentation outlined in the following section.

## 8. Knowledge Gaps and Priority Experimental Directions

The molecular components reviewed in Section 2, Section 3, Section 4, Section 5 and Section 6 vary widely in how firmly they are established. Table 3 classifies them by strength of evidence, species, and assay, distinguishing findings demonstrated experimentally in echinoids from those inferred by orthology or still untested. Classes were assigned by assay type rather than by the degree of confidence in a conclusion, according to three rules. First, each clause of an attributed function was classified separately, so that a single component may carry more than one class. Second, class A required a functional perturbation (morpholino, RNAi, or genome editing) or a biochemical assay (binding, enzymatic, promoter-activity, or transactivation) performed in an echinoid on the clause concerned; expression data of any kind, including in situ hybridization and immunolocalization, were classified as C. Third, class B required both a functional demonstration in another taxon and a documented ortholog in echinoids; where orthology is unresolved, the clause was classified as C or D. Classes were assigned by the authors; no formal inter-rater statistic was computed, and the rules are stated so that each assignment can be checked against the assay reported in the cited source. Table 4 summarizes the distribution of this evidence across species and study types: gene perturbation in the gonad has been reported only in *M. nudus* and *S. intermedius*, gene-expression data are concentrated in *M. nudus*, and several commercially exploited species, including *S. droebachiensis*, are represented only by manipulation, histological and compositional studies, whereas *L. albus* is represented only by gene-expression data. Read against that classification, the field’s unresolved points resolve into a consistent set of gaps.

### 8.1. A Consolidated Inventory of Molecular Gaps

The mechanistic account assembled across Section 2, Section 3, Section 4, Section 5 and Section 6 is uneven, and its unresolved points recur as a consistent set of gaps. Drawn together, they fall into three tiers. At the level of foundational biology, no experimentally validated gene-regulatory network for gonadal sex differentiation exists in any echinoid, the mode of sex determination has been mapped genomically only in *M. nudus*, the origin and lability of the gonadal primordium are uncharacterized, the gonadal piRNA repertoire has been surveyed only in the male gonad of *M. nudus*, and the adult-gonad methylome and chromatin-accessibility landscape have not been characterized. At the level of signaling, the biosynthetic route from retinol to retinoic acid has not been demonstrated in echinoderm tissue, the receptor mediating gonadal responses to exogenous steroids has not been identified, no functional link has been established between carotenoid metabolism and retinoid signaling, and the pathway transducing temperature and photoperiod into gonadal state remains unidentified in any echinoderm. At the level of applied translation, the enzymatic bioactivation of dietary substrates has not been mechanistically demonstrated, the transport of carotenoids into gonadal tissue remains inferred rather than shown, and the signals timing the accumulation-to-mobilization switch in nutritive phagocytes are unidentified beyond two candidate transcriptional regulators of *myp* acting within the somatic compartment. These gaps are not equally tractable, nor equally consequential for aquaculture; the purpose of this section is to distinguish those that current tools can close from those that must await new methods and to identify where targeted experimentation would yield the greatest return.

### 8.2. Ranking the Candidate Hypotheses

Not every gap constitutes a testable hypothesis with commercial relevance. Five candidate directions meet that dual criterion: they are experimentally falsifiable with existing tools and carry a plausible route to commercial return. Table 5 ranks them, prioritizing potential commercial return and using experimental falsifiability as the secondary criterion. Both criteria are ordinal judgments by the authors, not scores derived from a market or economic analysis, which lies outside the scope of this review. Commercial return was rated by how directly a direction acts on a variable that determines product value (GSI, color, sex) and by how firmly that link is documented; directions whose return is enabling or epistemic rather than product-level were rated as indirect or low. Falsifiability was rated by whether an interpretable test can be performed with established methods. The basis for each rating is stated in Table 5. The two axes do not covary, and the ranking makes that divergence explicit: some directions are readily testable yet commercially indirect, while others promise clear returns but rest on longer or less certain experimental chains. Read against the evidence classification of Table 3, the ranking also makes a third divergence visible: none of the five directions rests on tier-A evidence at the step that matters. The two highest-ranked are limited by the untested tier-D attribution of sexual-fate control to *Dmrt1* and *FoxL2* (H1) and by a tier-D mechanistic premise attached to otherwise well-established reserve biology (H2). This is the expected condition of a field in which descriptive resources have outpaced functional tests, and it is the reason these are framed as directions for experimentation rather than as recommendations for practice.

The two highest-ranked directions combine strong commercial return with immediate testability. Directed control of sex differentiation (H1) heads the ranking because female gonads can command a quality premium, although the evidence supports a narrower premium than a uniform preference. In *P. lividus*, consumers rated female gonads higher in color and acceptance in one feeding trial [172], but a study of wild gonads found no significant effect of sex on overall liking or acceptance [167], and a trained panel described male gonads as tending to be sweeter and female gonads as more intensely orange [168]. In *H. crassispina*, female gonads were lighter and more yellow, with stronger umami responses, whereas male gonads gave stronger sweetness and bitterness responses [171]. The premium therefore appears to rest mainly on color and appearance, and its size and consistency across species and markets remain to be established. The candidate regulators are identified and their expression characterized (Section 4), and adult gonadal RNAi has already been demonstrated in *M. nudus* and in *S. intermedius*, where knockdown of the testis-expressed gene *spata4* lowered *Dmrt1* and germ-cell marker expression [8], making perturbation feasible with existing methods. A knockdown that fails to shift sexual phenotype would, however, be interpretable only if depletion is quantified, a positive control shows that the delivery route perturbs gonadal gene expression, and, ideally, the phenotype is rescued; without these controls, a null result could reflect incomplete knockdown, compensation, redundancy, a fate already fixed, or limits of delivery. In adult testes of the sea cucumber *A. japonicus*, *Dmrt1* knockdown altered F*oxL2* and *SoxE* expression without detectable histological change [101], showing that a molecular response need not produce a phenotype over the timescale tested. Dietary and enzymatic modulation of gonad yield (H2) ranks second: gonad index is the central commercial variable, the readouts (RNA-seq, histology, MYP quantification) are routine, and the approach integrates directly into existing formulation practice, though its mechanistic premise remains to be demonstrated.

The remaining three directions each rank lower for a distinct reason, and together they illustrate why falsifiability and return must be weighed separately. The carotenoid-to-retinoid axis (H3) offers an attractive return through pigmentation and possible maturation control, but its experimental chain is long: pigment cleavage, ligand generation, receptor engagement, and transcriptional output must each be demonstrated, and an ambiguous result at any step fails to localize the break. The cross-family generality of the sex-determination system (H4) is highly tractable, because female heterogamety is already mapped to a single chromosomal region in *M. nudus* and provides a reference against which the cytogenetic inference of male heterogamety in *P. lividus* can be tested directly. Its return is indirect, enabling the directed sex control (H1) rather than delivering a product outcome itself. Endocrine modulation as a functional probe (H5) is the clearest case of the two axes diverging: exposure studies already alter steroid ratios and reproduction-related genes such as *cyp17* and *myp* in predictable directions [166,170], making it highly falsifiable, yet its value is epistemic rather than applied—endocrine disruptors are contaminants, not viable culture inputs, and their utility lies in mapping the steroidogenic pathway that Section 5 left unresolved.

### 8.3. Where to Begin

Following the ranking directly, the two leading directions combine the highest commercial return with present-day testability and are those for which a first experiment can be designed with existing methods. Directed sex control (H1) offers the strongest combination: a documented market preference for female gonads based mainly on color, characterized candidate regulators, and available perturbation tools. Dietary and enzymatic modulation of yield (H2) is an equally testable and commercially central complement, addressing the gonad index that underpins the industry. The three lower-ranked directions retain clear scientific value and would strengthen the mechanistic foundation, but by the criterion adopted here, they follow rather than precede the two leading candidates. The resulting order defines a priority for experimentation, not a recommendation for practice, consistent with the evidence status of each direction (Section 8.2).

Translation of a positive H1 result into production-scale sex control faces constraints of a different order. RNAi- and morpholino-mediated effects in the adult gonad are non-heritable and would require treatment of each individual, whereas stable edited lines depend on rearing CRISPR/Cas9-injected embryos through metamorphosis to reproductive adulthood [158]. In finfish and crustacean aquaculture, monosex stocks are generally obtained by sex-reversing broodstock into neo-males or neo-females and crossing them within an established genetic sex system, the element that remains unresolved for most echinoids (H4). A production route therefore presupposes three conditions: a characterized sex-determination system in the target species, a reliable genetic sex marker, and a reversal treatment whose effect persists to reproduction. Sex-linked markers are available for *M. nudus* [9,64] and *S. intermedius*, in which phenotypic and genetic sex were discordant in a subset of individuals, although dietary estradiol did not reverse phenotypic sex in males over 58 days [8]; we found no report of experimentally induced sex reversal in an echinoid. Gene-editing routes would additionally fall under regulatory frameworks for edited food animals that vary among producing countries, and consumer acceptance of such products remains to be assessed. Table 6 sets out these directions as concrete experiments, specifying for each a target species, technique, measurable endpoint, and bench feasibility with currently available tools.

## 9. Conclusions

The molecular biology of the echinoid gonad has reached a point at which several long-standing uncertainties are no longer merely unresolved but decidable. Each hinges on an experiment that existing tools can perform and whose outcome would discriminate between hypotheses that currently coexist. Two of these questions combine commercial return with immediate testability and should be addressed first; two further questions rank below them on return but would settle premises on which the first two depend.

The first is whether sexual fate remains modifiable once set. Dimorphic expression of *Dmrt1* and *FoxL2* establishes correlation, not control; the distinction is resolvable by perturbation, now feasible in adult gonadal tissue. If knockdown shifts gonadal phenotype toward the opposite program, sexual fate is regulatively labile and directed sex control becomes tractable. If a quantified and controlled knockdown does not, the most direct reading is that sex is fixed upstream of these regulators or before the stage tested.

The second is whether gonad yield can be raised through a defined molecular route rather than by empirical formulation alone. Gonad index is largely a function of nutritive-phagocyte mass and its stored reserves, and the readouts are routine. A controlled feeding trial comparing diets that differ only in a candidate bioactive substrate would test whether reserve loading responds to a specific dietary input and whether that response carries a transcriptional signature in the nutritive-phagocyte compartment. A positive result would give the industry its first mechanistically anchored feed variable; a null result would return attention to the regulators of nutritive-phagocyte accumulation and mobilization that remain unidentified beyond two candidate regulators of *myp*.

Two enabling questions follow. The mode of sex determination appears to differ between the two species examined: male heterogamety rests on a cytogenetic inference from a single karyotype study in one, whereas female heterogamety has been mapped to a single chromosomal region in the other. Sex-linkage mapping in a second echinoid family would establish whether the determination system is conserved across echinoids and would fix the upstream boundary against which any result of the first experiment must be read. The retinoid axis, in turn, is either an endogenous signaling system or only a route of response to dietary retinoids. Echinoids possess receptors, catabolic machinery, and response elements but lack a demonstrated biosynthetic route from retinol to retinoic acid; identifying the enzyme that substitutes for the absent ALDH1A would either establish retinoic acid as a genuine internal regulator or reduce the axis to a means of responding to dietary intake, on which every downstream inference currently depends.

The four lines of work synthesized in this review have developed largely in parallel, addressed by separate research communities and rarely considered together. Their integration into a single mechanistic account is intended to serve as a working reference: one that distinguishes what has been experimentally established from what is inferred by homology and what remains untested and that traces how findings in developmental genetics, gonadal transcriptomics, germ-cell biology, and endocrine signaling bear on one another.

## Figures and Tables

**Figure 1:**
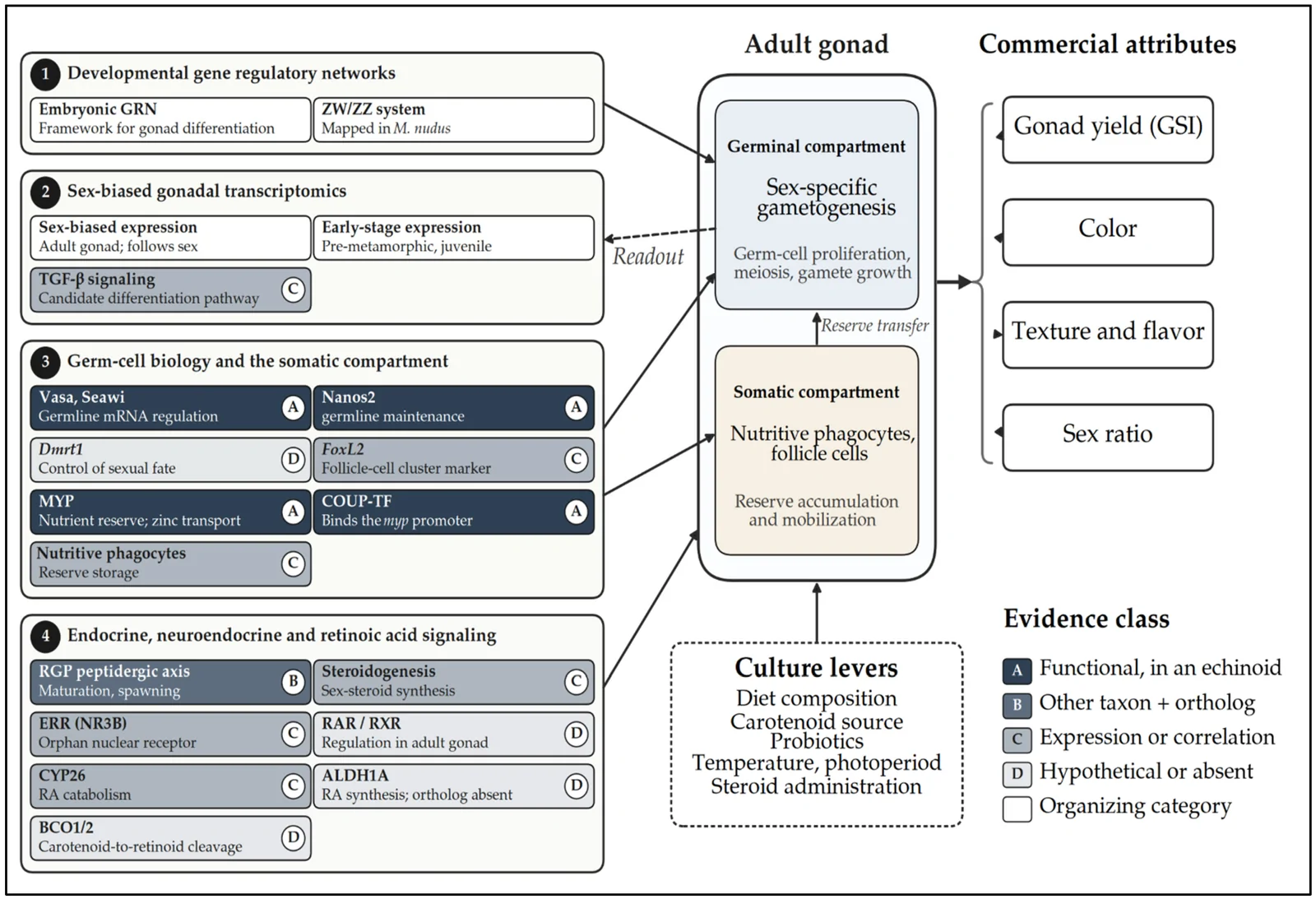
Organization of the evidence reviewed around the adult echinoid gonad. Four lines of evidence (1–4) are mapped onto the germinal and somatic compartments of the gonad, and the gonad onto the commercial attributes of the product; culture levers act on the gonad as a whole. Each molecular component is shaded by the class of evidence for the function indicated (classes A–D, defined in Section 1; assignment rules in Section 8); unshaded elements are organizing categories that were not classified. Arrows indicate the compartment on which each line of evidence bears, not demonstrated regulatory interactions; the dashed arrow indicates that sex-biased expression in the adult gonad is a readout of sex rather than evidence on its determination (Section 4). The scheme organizes the evidence by control point and does not represent a unified mechanistic model. Shading refers to evidence class and differs from the evidence-status colors of Figure 2 and Figure 3, which indicate where a component has been demonstrated. Abbreviations: ALDH1A, aldehyde dehydrogenase 1A; BCO, β-carotene oxygenase; *COUP-TF*, chicken ovalbumin upstream promoter–transcription factor; CYP26, cytochrome P450 family 26; ERR, estrogen-related receptor; GRN, gene regulatory network; GSI, gonadosomatic index; MYP, major yolk protein; RA, retinoic acid; RAR, retinoic acid receptor; RGP, relaxin-like gonad-stimulating peptide; RXR, retinoid X receptor; TGF-β, transforming growth factor β. Rendered in Python (3.14.4).

**Figure 2:**
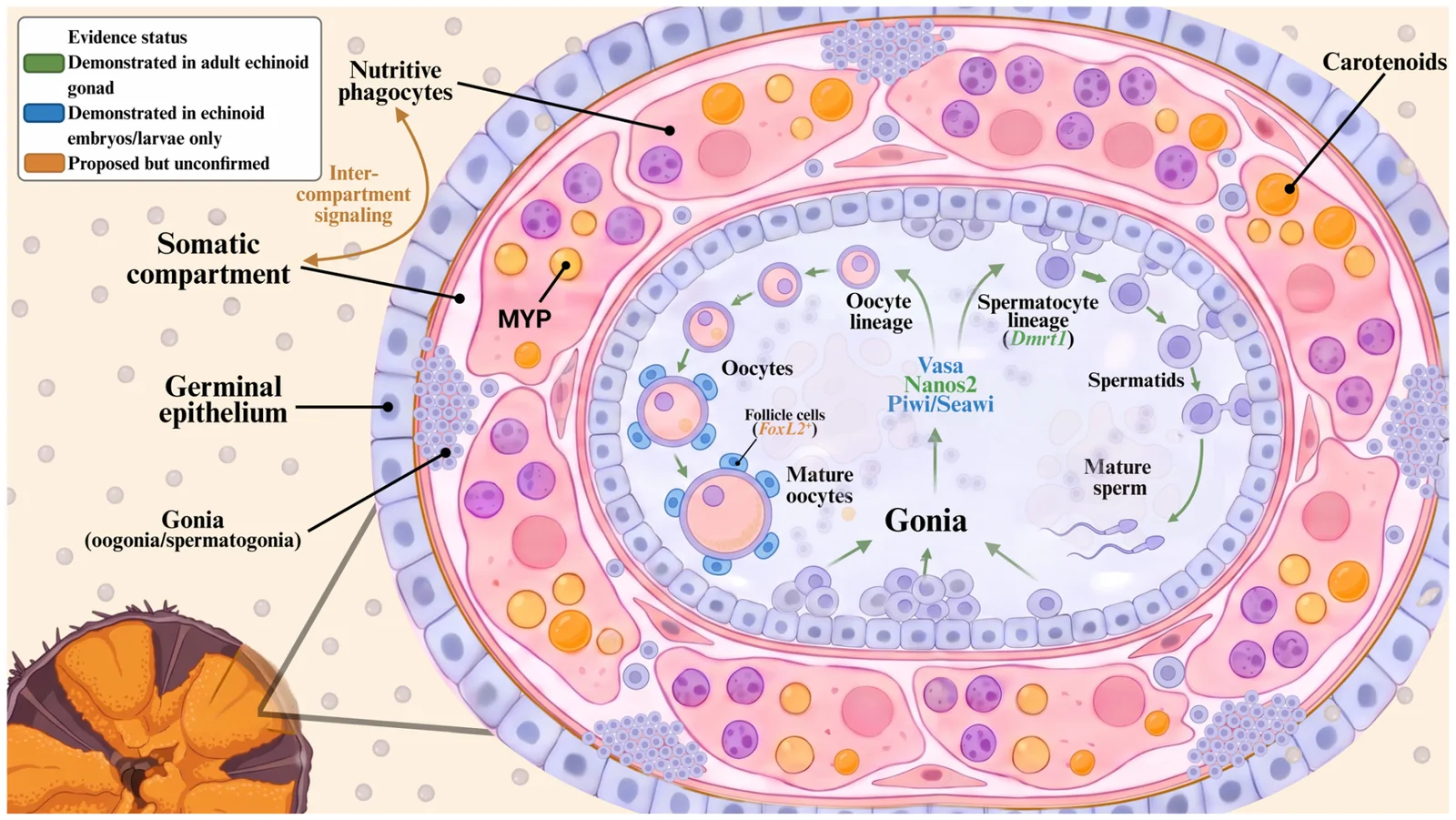
Dual-compartment architecture of the euechinoid gonad and the evidence status of its molecular components. The acinus is bounded by the germinal epithelium; interior to it lies the somatic compartment, containing nutritive phagocytes that store the major yolk protein (MYP) and carotenoid-bearing lipid droplets, together with follicle cells associated with developing oocytes, whose identification with a *FoxL2*-enriched single-cell cluster remains inferential. Gonia, descended from primordial germ cells of the small micromere lineage, line the germinal epithelium and give rise to the oocyte and spermatocyte lineages which are drawn within a single acinus for comparison; in gonochoristic echinoids each gonad is either an ovary or a testis. The germ-line factors Vasa, Nanos2, and Piwi/Seawi are shown across the lineage; their expression and function have been characterized mainly in embryos and larvae, and in the adult gonad only for Nanos2 in *M. nudus. Dmrt1* marks the male lineage. In the intact tissue, gametogenesis proceeds within the nutritive phagocyte compartment, each gamete enclosed by phagocyte cytoplasm across which MYP reserves are transferred; for clarity, the gametogenic sequences are drawn here as a central zone whose boundary is schematic rather than anatomical. Follicle cells are drawn only around oocytes because the single-cell data derive from ovaries; somatic cell types of the testis other than nutritive phagocytes remain unresolved. Arrows indicate the progression of germ-cell differentiation from gonia; the double-headed arrow indicates proposed signaling between compartments. Green elements denote molecular components demonstrated in the adult echinoid gonad; blue, in echinoid embryos or larvae only; orange, proposed but unconfirmed. Created in BioRender. Rodríguez, P. (2026) https://BioRender.com/la1adjl (accessed on 25 September 2026).

**Table 1:** Exogenous steroid and steroid-active exposures in echinoids. Studies also differ in dose, life stage and initial gonadal stage, which limits comparison among species. ↑, increase; ↓, decrease.

Species	Compound	Route; Duration	Gonadal Outcome	Ref.
***P. depressus***	Androstenedione, estrone, estradiol-17β, testosterone, progesterone	Dietary; 30 d	Juvenile males: gonad index ↑ (androstenedione, estrone), spermatogenesis ↑ (estrone); estradiol-17β no effect; females unaffected	[118]
***L. variegatus***	Estradiol, progesterone, testosterone	Dietary; 36 d	Progestins: reserve accumulation ↑, testis growth ↑; estradiol: ovary index ↑, oocyte diameter ↓; testosterone: oocyte growth ↑	[116]
***P. lividus***	Estradiol	Peristomial injection; 2 and 12 wk	No marked effect; gonad index tracked feed intake	[120]
***P. lividus***	Estradiol, testosterone	Dietary; ovarian cell culture	No effect on gonad or gamete growth; MYP unchanged in vitro	[117]
***S. intermedius***	Estradiol	Dietary; 58 d	Adult males: sex-related gene expression altered; no histological sex reversal	[8]
***P. lividus***	Ethinylestradiol (synthetic)	Waterborne; 7 and 28 d	Females: maturation ↑ at 28 d; males: more low-development stages at 7 d	[122]
***P. lividus***	Triphenyltin (steroid-metabolism disruptor)	Waterborne; 4 wk	Aromatase ↓; 5α-reductase and testosterone conjugation ↑; gonadal testosterone stable	[123]

**Table 2:** Molecular control points mapped to aquaculture objectives in euechinoids. For each production objective, the governing mechanism(s), the species in which those mechanisms have been examined experimentally, the current state of knowledge, and the control lever the mechanism might afford. Confidence is uneven across rows: some levers rest on well-characterized biology, others remain contingent on demonstrations not yet produced. Abbreviations: EBP, echinenone-binding protein.

Aquaculture Objective	Governing Mechanism(s)	Species with Experimental Evidence	Current Knowledge Status	Proposed Control Lever
**Broodstock conditioning**	Germ-line GRN (Vasa/Piwi/Nanos2); sex differentiation (*Dmrt1*/*FoxL2*)	*S. purpuratus* (Vasa, Seawi); *M. nudus* (Nanos2, *Dmrt1*, *FoxL2*); *L. albus* (*Dmrt1*/*FoxL2*); *S. intermedius* (sex-related genes)	Candidate markers described (*nanos2*, *Dmrt1*); *FoxL2* sex-bias unresolved between species; no experimentally validated gonadal sex-differentiation GRN; sex-determination system characterized only in *M. nudus* (ZW/ZZ).	Marker-guided conditioning (*nanos2*/*Dmrt1*) to synchronize maturation and spawning; sex-differentiation regulators as targets for directed sex ratios
**Gonad yield/roe enhancement (elevated GSI)**	Nutritive phagocytes; MYP (intestinal + gonadal synthesis); reserve cycling	*S. purpuratus*, *L. variegatus* (single-cell ovarian atlas); *S. droebachiensis*, *P. lividus*, *M. nudus*, *L. albus* (bipartite architecture); *P. depressus* (MYP two-site synthesis); *S. purpuratus*, *P. lividus*, *M. nudus* (MYP characterization)	MYP dynamics and reserve cycling well characterized; empirical diet formulation established; enzymatic bioactivation of dietary substrates not mechanistically demonstrated	Diet formulation targeting nutritive-phagocyte reserve loading; probiotic-mediated bioactivation of dietary substrates to enhance MYP accumulation and gonad index
**Roe quality/pigmentation**	Carotenoid metabolism; retinoid axis (RAR/RXR, CYP26); carotenoid transport (EBP/FABP-like, lipoprotein route)	*S. intermedius* (LDL-linked coloration); *P. depressus* (intestinal bioconversion); *E. chloroticus* (echinenone-binding protein); *S. purpuratus* (RA machinery); commercial euechinoids (carotenoid profiles)	Carotenoid accumulation and intestinal bioconversion well described; retinoid-axis machinery present but RA biosynthesis undemonstrated; carotenoid-to-retinoid conversion and receptor function untested	Dietary carotenoid supplementation for pigmentation; enzymatic carotenoid cleavage to retinoid ligands as one candidate axis linking pigment intake to gonadal maturation
**Seed/juvenile production**	Developmental GRNs (Section 2.1 and Section 2.2); larva-to-gonad transition	*S. purpuratus* (embryonic/larval GRN); *H. erythrogramma* (developmental-mode rewiring); *P. lividus*, and *S. purpuratus* (gene-activation timing)	Embryonic/larval GRN exceptionally resolved; larva-to-gonad transition and primordium origin poorly characterized; no direct link from developmental regulators to hatchery performance established	Settlement and metamorphosis cues informed by developmental GRN knowledge; early-stage signaling as a target for improving seed survival and quality

**Table 3:** Evidence classification for the principal molecular components of echinoid gonadal biology. For each component, the attributed function, the strength of the supporting evidence, the species in which it was obtained, and the assay or basis are given. Evidence classes: **A** = experimentally demonstrated in an echinoid (functional perturbation or biochemical assay); **B** = demonstrated in another taxon and inferred by orthology; **C** = plausible inference from expression, genomic presence, or correlation; **D** = hypothetical or undemonstrated (including documented absence). Where two classes are given, they correspond in order to the clauses of the attributed function. Classes are assigned by the rules stated in Section 8. Species names are abbreviated as in the text. Abbreviations: CYP, cytochrome P450; EMSA, electrophoretic mobility shift assay; ISH, in situ hybridization; MS, mass spectrometry; PGC, primordial germ cell; RARE, retinoic acid response element; RNAi, RNA interference; RT-PCR, reverse-transcription PCR; RT-qPCR, quantitative reverse-transcription PCR.

Molecular Component	Attributed Function	Evidence	Species (Evidence)	Assay/Basis
Germ line				
Vasa	Translational regulation of germ-line mRNAs; *cyclinB* translation and cell-cycle progression	**A**	*S. purpuratus*	Morpholino knockdown; translational/biochemical assays
Seawi (Piwi)	PGC survival; regulation of germ-line mRNAs (*vasa*, *gustavus*)	**A**	*S. purpuratus*	Morpholino knockdown
Nanos2	Germ-line maintenance; germ-cell marker (ovary)	**A/C**	*M. nudus* (adult); *S. purpuratus* (embryo)	RNAi knockdown; in situ hybridization (ISH); RT-qPCR
Sex differentiation				
*Dmrt1*	Testis-specific expression in spermatocytes and spermatogonia; promoter regulatory regions; control of gonadal sexual fate	**C/A**/**D**	*M. nudus*; (expression, promoter); none (sexual fate)	RT-qPCR; ISH; immunolocalization; promoter activity assay; Dsx-like class, no vertebrate *Dmrt1* ortholog
*FoxL2*	Marker of somatic cluster assigned to follicle cells; direction of gonadal sex bias unresolved between species	**C**	*S. purpuratus*, *L. variegatus* (cluster); *P. miniata* (follicle cells)	Single-cell transcriptomics; in situ hybridization (asteroid); RT-qPCR
TGF-β signaling	Candidate gonadal-differentiation pathway	**C**	*M. nudus*	Transcriptomics (sex-biased expression)
Somatic compartment				
MYP	Nutrient reserve; zinc transport from gut to gonad	**A**	*P. depressus*	Zinc-binding assays; labeled-protein tracing
COUP-TF	Binding and transactivation of *myp* promoter; regulator of gonadal *myp*	**A/C**	*M. nudus*	Promoter cloning; EMSA; transactivation assay; expression correlation
Nutritive phagocyte	Somatic reserve storage; correspondence to single-cell clusters	**C/D**	*S. droebachiensis* (histology); *S. purpuratus* (correspondence untested)	Histology; cytochemistry; single-cell transcriptomics
Endocrine/neuroendocrine				
ERR (NR3B)	Orphan nuclear receptor; candidate reproductive regulator	**C**	*S. purpuratus* (genomic); *M. nudus* (male-biased gonadal expression)	Phylogenomic identification; gonadal expression (RT-PCR and RT-qPCR)
RGP/peptidergic axis	Peptide gonadotropin; maturation and spawning induction	**B**	Asteroids, holothurians (function); *S. purpuratus* (complement present)	Synthetic-peptide bioassay (other classes); peptidomics (echinoid)
Steroidogenesis (CYP pathway)	Sex-steroid synthesis (*cyp19A* absent; route unresolved)	**C**	*S. purpuratus* (genomic); *M. nudus* (gonadal expression); various (steroid detection)	Phylogenomics; RT-qPCR; steroid immunoassay/MS
Retinoid signaling				
RAR/RXR	Embryonic patterning and RA binding; transcriptional regulation in adult gonad	**A**/**B/D**	*M. nudus* (RXR, embryo); *P. dumerilii*, *P. caudatus* (RAR affinity)	Morpholino knockdown (embryo); in vitro binding in *P. dumerilii* and *P. caudatus*
CYP26	RA catabolism; retinoid-response readout	**C**	Echinoderms (gene presence); *S. purpuratus* (RAREs)	Comparative genomics; sequence and RARE analysis
ALDH1A	RA biosynthesis (ortholog absent in echinozoans)	**D**	*S. purpuratus*, *A. japonicus* (absence)	Comparative genomics (documented absence)
Carotenoid cleavage (BCO1/2)	Carotenoid-to-retinoid conversion (hypothetical)	**D**	*S. purpuratus* (single annotation, uncharacterized); molluscs (comparative)	Genome annotation only; BCO2 functionally characterized in *P. yessoensis*

**Table 4:** Distribution of primary gonadal evidence across echinoid species and study types. Each reference is listed under every study type it reports; reviews, genome assemblies, sex-marker genotyping and studies restricted to embryos or larvae are excluded. –, no study identified.

Species	Gene Expression	Gene Perturbation	Dietary, Hormonal or Environmental Manipulation	Histology and Staging	Biochemical Composition
*P. lividus*	[21,147]	–	[2,15,117,120,122,123,147,162,165,166]	[15,70,105,122,166]	[85,105,117,123,147,165,166,167,168]
*M. nudus*	[25,26,28,29,30,79,82,98,99,107,110]	[28]	[5]	[25,28,110]	[25,99]
*S. intermedius*	[8,24,67]	[8]	[8,16,161,163,169]	[16,67]	[16,163]
*S. purpuratus*	[23,65]	–	[4,11,76]	[23,69]	[4,11,76]
*L. variegatus*	[22,65,66]	–	[116,119]	[66]	[116,119]
*P. depressus*	[71,96]	–	[118]	[118]	[86,96,97]
*H. crassispina*	[170]	–	[170]	[170]	[150,170,171]
*S. droebachiensis*	–	–	[6,78]	[32]	[6,78]
*L. albus*	[20,103]	–	–	–	–
Other echinoids	–	–	*P. miliaris* [151]; *E. esculentus* [6]; *T. gratilla* [77]	*D. excentricus* [83]	*P. miliaris* [151,152]; *E. esculentus* [6]; *D. setosum*, *S. sphaeroides* [150]; *E. chloroticus* [155]

**Table 5:** Candidate research directions ranked by potential commercial return (primary criterion) and experimental falsifiability (secondary). The two axes do not covary: some directions are readily testable yet commercially indirect (H4, H5), while others promise clearer returns on longer experimental chains (H3). Ratings are ordinal judgments by the authors against the criteria stated in Section 8.2; no quantitative scoring was applied. References [1,4], cited for H2, are by authors of this review. Abbreviations: RNA-seq, RNA sequencing.

Candidate Direction	Commercial Return	Experimental Falsifiability	Key References
Directed control of sex differentiation (female-biased production)	High: female gonads preferred mainly for color; sensory and market premium varies by species and study	Medium: controlled knockdown discriminates regulatory control from correlated expression (quantified depletion, positive control, rescue); null results interpretable with these controls	[20,29,64,79,95,158,167,172]
Dietary and enzymatic modulation of gonad yield	High: gonad index is the central commercial variable; integrates into existing formulation practice	High: RNA-seq, histology, and MYP quantification; mechanistic premise still to be demonstrated	[1,4,14,30]
Carotenoid-to-retinoid signaling axis	Medium–high: direct return through pigmentation; maturation link candidate	Medium: long experimental chain (cleavage → ligand → receptor → output); ambiguous results do not localize the break	[24,36,155,156,157]
Cross-family generality of the sex-determination system	Indirect: enables directed sex control (H1) rather than delivering a product outcome	High: genomic sex-linkage mapping in *P. lividus* and *M. franciscanus* tests the XY inference and its concordance with the *M. nudus* chromosome 7 region, using existing sequencing tools	[9,61,63,64]
Endocrine modulation as a functional probe	Low/epistemic: endocrine disruptors are contaminants, not culture inputs; value lies in mapping the steroidogenic pathway	High: exposure studies already shift steroid ratios and reproduction-related genes (*cyp17*, *myp*) in predictable directions	[107,108,166,170]

**Table 6:** Operational experimental agenda for the priority directions and aquaculture-relevant gaps. For each, a concrete experiment is specified by target species, technique, measurable endpoint, and feasibility with currently available tools. The five ranked candidate directions (H1–H5; Table 5) are listed first, followed by descriptive gaps of direct relevance to production. Feasibility reflects the readiness of existing methods, not the certainty of a positive result or feasibility at production scale. Abbreviations: 2b-RAD, type IIB restriction-site-associated DNA sequencing; CRISPR/Cas9, clustered regularly interspaced short palindromic repeats/CRISPR-associated protein 9; GWAS, genome-wide association study; LC–MS/MS, liquid chromatography–tandem mass spectrometry.

Direction/Gap	Target Species	Technique	Measurable Endpoint	Bench Feasibility with Current Tools
H1—Directed sex control	*M. nudus*, *S. purpuratus*	RNAi, morpholino, or CRISPR/Cas9 knockdown of *Dmrt1*/*FoxL2*	Shift in gonadal sexual phenotype (histology; sex-marker expression)	Medium—embryonic perturbation established; adult gonadal RNAi demonstrated (*M. nudus nanos2*; *S. intermedius spata4*); knockout adults raised from CRISPR/Cas9-injected embryos (*H. pulcherrimus*); adult *Dmrt1*/*FoxL2* knockdown not yet reported; production use requires H4
H2—Dietary/enzymatic yield modulation	*S. purpuratus*, *S. intermedius*	Controlled feeding trial with substrate-bioactivating vs. non-bioactivating diets; RNA-seq + histology	Gonad index; MYP accumulation; gametogenic-stage distribution	High—feeding trials, transcriptomics, and MYP quantification are routine
H3—Carotenoid-to-retinoid axis	*S. intermedius*, *S. purpuratus*	BCO functional assay of *LOC752114*; retinoid quantification (LC–MS/MS); CYP26 induction readout	Retinoid ligand generation; CYP26 response; receptor engagement	Medium—long chain; each step assayable but ambiguous results do not localize the break
H4—Cross-family generality of sex determination	*P. lividus*, *M. franciscanus*	Genomic sex-linkage mapping (2b-RAD sequencing, genome-wide association study (GWAS), assembly)	Female- vs. male-specific linkage signal (ZW vs. XY); synteny with the *M. nudus* chromosome 7 region	High—achievable with existing sequencing and mapping
H5—Endocrine modulation as functional probe	*P. lividus*, *H. crassispina*	Controlled endocrine-disrupting compound (EDC) exposure; targeted gene/steroid readout	Change in *cyp17*/*myp* expression; steroid ratios	High—exposure protocols and readouts established (research use only)
Nutritive-phagocyte regulation (yield)	*S. purpuratus*, *P. depressus*	Stage-resolved transcriptomics + histology across the reproductive cycle	Temporal MYP/lipogenesis expression; accumulation-to-mobilization timing	High—sampling and profiling feasible; interpretation limited by staging resolution
Neuroendocrine spawning axis (RGP)	*S. purpuratus*	Ortholog identification + synthetic-peptide maturation bioassay	Oocyte maturation/spawning induction ex vivo	Medium—bioassay established in other classes; echinoid ortholog not yet identified
Validated gonadal sex-differentiation GRN	*M. nudus*, *S. purpuratus*	Systematic perturbation of candidate regulators with network readout	Regulatory dependencies among *Dmrt1*, *FoxL2*, and partners	Medium—tools available; scale of systematic perturbation is the constraint
Environmental transduction	*S. intermedius*, *P. lividus*	Controlled photothermal regime with stage-matched gonadal RNA-seq	Differential expression under contrasting regimes at matched gametogenic stage	High—regimes and transcriptomics both established

## Data Availability

No new data were created or analyzed in this study. Data sharing is not applicable to this article.

## Abbreviations

ALDH1A	Aldehyde dehydrogenase 1A
BCO	β-carotene oxygenase
COUP-TF	Chicken ovalbumin upstream promoter-transcription factor
CRABP	Cellular retinoic acid-binding protein
CRISPR	Clustered regularly interspaced short palindromic repeats
CYP	Cytochrome P450
CYP26	Cytochrome P450 family 26
EBP	Echinenone-binding protein
EDC	Endocrine-disrupting compound
ERR	Estrogen-related receptor
GRN	Gene regulatory network
GSI	Gonadosomatic index
GWAS	Genome-wide association study
HNF4	Hepatocyte nuclear factor 4
ISH	In situ hybridization
LDL	Low-density lipoprotein
MYP	Major yolk protein
NR	Nuclear receptor
PGC	Primordial germ cell
piRNA	PIWI-interacting RNA
PPAR	Peroxisome proliferator-activated receptor
RA	Retinoic acid
RAR	Retinoic acid receptor
RARE	Retinoic acid response element
RGP	Relaxin-like gonad-stimulating peptide
RNAi	RNA interference
RXR	Retinoid X receptor
SR-B1	Scavenger receptor class B type 1
TGF-β	Transforming growth factor beta
